# Empirical Evaluation of Voluntarily Activatable Muscle Synergies

**DOI:** 10.3389/fncom.2017.00082

**Published:** 2017-09-06

**Authors:** Shunta Togo, Hiroshi Imamizu

**Affiliations:** ^1^Graduate School of Informatics and Engineering, The University of Electro-Communications Tokyo, Japan; ^2^Cognitive Mechanisms Laboratories, Advanced Telecommunications Research Institute International Kyoto, Japan; ^3^Department of Psychology, The University of Tokyo Tokyo, Japan

**Keywords:** muscle synergies, non-negative matrix factorization, isometric force production, electromyography, motor primitive

## Abstract

The muscle synergy hypothesis assumes that individual muscle synergies are independent of each other and voluntarily controllable. However, this assumption has not been empirically tested. This study tested if human subjects can voluntarily activate individual muscle synergies extracted by non-negative matrix factorization (NMF), the standard mathematical method for synergy extraction. We defined the activation of a single muscle synergy as the generation of a muscle activity pattern vector parallel to the single muscle synergy vector. Subjects performed an isometric force production task with their right hand, and the 13 muscle activity patterns associated with their elbow and shoulder movements were measured. We extracted muscle synergies during the task using electromyogram (EMG) data and the NMF method with varied numbers of muscle synergies. The number (*N*) of muscle synergies was determined by using the variability accounted for (VAF, *N*_*VAF*_) and the coefficient of determination (CD, *N*_*CD*_). An additional muscle synergy model with *N*_*AD*_ was also considered. We defined a conventional muscle synergy as the muscle synergy extracted by the *N*_*VAF*_, *N*_*CD*_, and *N*_*AD*_. We also defined an extended muscle synergy as the muscle synergy extracted by the *N*_*EX*_> *N*_*AD*_. To examine whether the individual muscle synergy was voluntarily activatable or not, we calculated the index of independent activation, which reflects similarities between a selected single muscle synergy and the current muscle activation pattern of the subject. Subjects were visually feed-backed the index of independent activation, then instructed to generate muscle activity patterns similar to the conventional and extended muscle synergies. As a result, an average of 90.8% of the muscle synergy extracted by the *N*_*VAF*_ was independently activated. However, the proportion of activatable muscle synergies extracted by *N*_*CD*_ and *N*_*AD*_ was lower. These results partly support the assumption of the muscle synergy hypothesis, i.e., that the conventional method can extract voluntarily and independently activatable muscle synergies by using the appropriate index of reconstruction. Moreover, an average of 25.5% of the extended muscle synergy was significantly activatable. This result suggests that the CNS can use extended muscle synergies to perform voluntary movements.

## Introduction

Humans must control multiple joints to perform a single motor task. Since one joint is moved by multiple muscles, the same joint movements can be achieved by a variety of muscle activity pattern combinations. Therefore, the central nervous system (CNS) must select a specific muscle activity pattern from infinite combinations to achieve the task. This redundancy problem, first proposed by Bernstein (1967), is still an important unresolved puzzle. To approach the problem of redundancy in muscle control, the muscle synergy hypothesis is attracting attention (Tresch et al., 1999; Saltiel et al., 2001; d'Avella et al., 2003; Bizzi et al., 2008; d'Avella and Lacquaniti, 2013). Muscle synergy is defined as a neurophysiological control module dominating multiple muscles. In the muscle synergy hypothesis, the CNS controls high degrees of freedom (DOF) muscles via low DOF muscle synergies. The muscle synergy hypothesis has been supported by many experimental results. For example, small sets of muscle synergies could robustly reconstruct the original muscle activation patterns of various movements (upper limb movements: Weiss and Flanders, 2004; d'Avella et al., 2006; lower limb movements: Ivanenko et al., 2007; Hug et al., 2011; Chvatal and Ting, 2012; and isometric force production: de Rugy et al., 2013; Berger and d'Avella, 2014). Furthermore, similar muscle synergies were adopted in similar movements (Cappellini et al., 2006), and muscle synergies were robustly preserved for changes in task parameters (d'Avella et al., 2008; Gentner et al., 2013). In addition to human examples, studies of movements in non-human primates (Overduin et al., 2008, 2015), cats (Ting and Macpherson, 2005; Torres-Oviedo et al., 2006), and frogs (Tresch et al., 1999; d'Avella et al., 2003) also suggested a control mechanism reflecting the muscle synergy hypothesis. Therefore, the muscle synergy hypothesis is a major hypothesis that can explain the neurophysiological mechanisms of multi-muscle control by the CNS in vertebrates. In the muscle synergy hypothesis, there is a tradeoff between the complexity of muscle synergies and the number of muscle synergies required to encode a motor skill. The conventional method determines the minimum number of muscle synergies required to sufficiently represent the motor skill (original muscle activity patterns). The CNS serially activates muscle synergies—patterns of muscle activation—to implement a motor skill.

The time-invariant muscle synergy model explains time profiles of measured muscle activities (electromyogram: EMG) ***m***(*t*) ∈ℝ^*M*^ by combining muscle synergy (spatial pattern of muscle coordination) ***w*** ∈ℝ^*M*^ and the coefficient of activation (time profile of motor command) *c*(*t*) as ***m***(*t*) = $\sum_{i=1}^{N}c_{i}\left(t\right){\text{w}}_{\text{i}}$ (Ivanenko et al., 2003; Alessandro et al., 2013). Muscle synergy vector ***w*** indicates weighting coefficient of muscle activation for a motor command *c*(*t*). The CNS achieves the motor task by applying the appropriate motor command *c*(*t*) to muscle synergy ***w***. Therefore, the muscle synergy model assumes that an individual muscle synergy is a control module that can be independently and voluntarily activated. However, this important assumption has not been experimentally tested, to our knowledge. Some earlier studies reported experimental results suggesting hierarchical multi-muscle control similar to a muscle synergy model (de Rugy et al., 2012; Berger et al., 2013). However, it is not clear whether the extracted individual muscle synergy is an independently and voluntarily activatable control module or a mathematical artifact. If the extracted muscle synergy cannot be independently activated, there is a possibility that the original movement is encoded by factors other than the extracted muscle synergies. In such a case, the muscle synergies that could not be voluntarily and independently activated would be deemed mathematical artifacts or involuntarily activated modules. In this study, we experimentally evaluated voluntary activation of individual muscle synergies to test the hypothesis that the conventional method can extract voluntarily activatable muscle synergies. If subjects could voluntarily activate one extracted muscle synergy (one motor command could be set as *c*(*t*) = 1, and the other motor commands could be set as *c*(*t*) = 0), i.e., subjects could produce a muscle activity pattern vector parallel to the one muscle synergy vector (***m***(*t*) = ***w***), there would be an independently activatable neural circuit representing extracted muscle synergy. Therefore, we can experimentally evaluate whether the extracted muscle synergy is independently and voluntarily controllable module or not.

**Figure 1:**
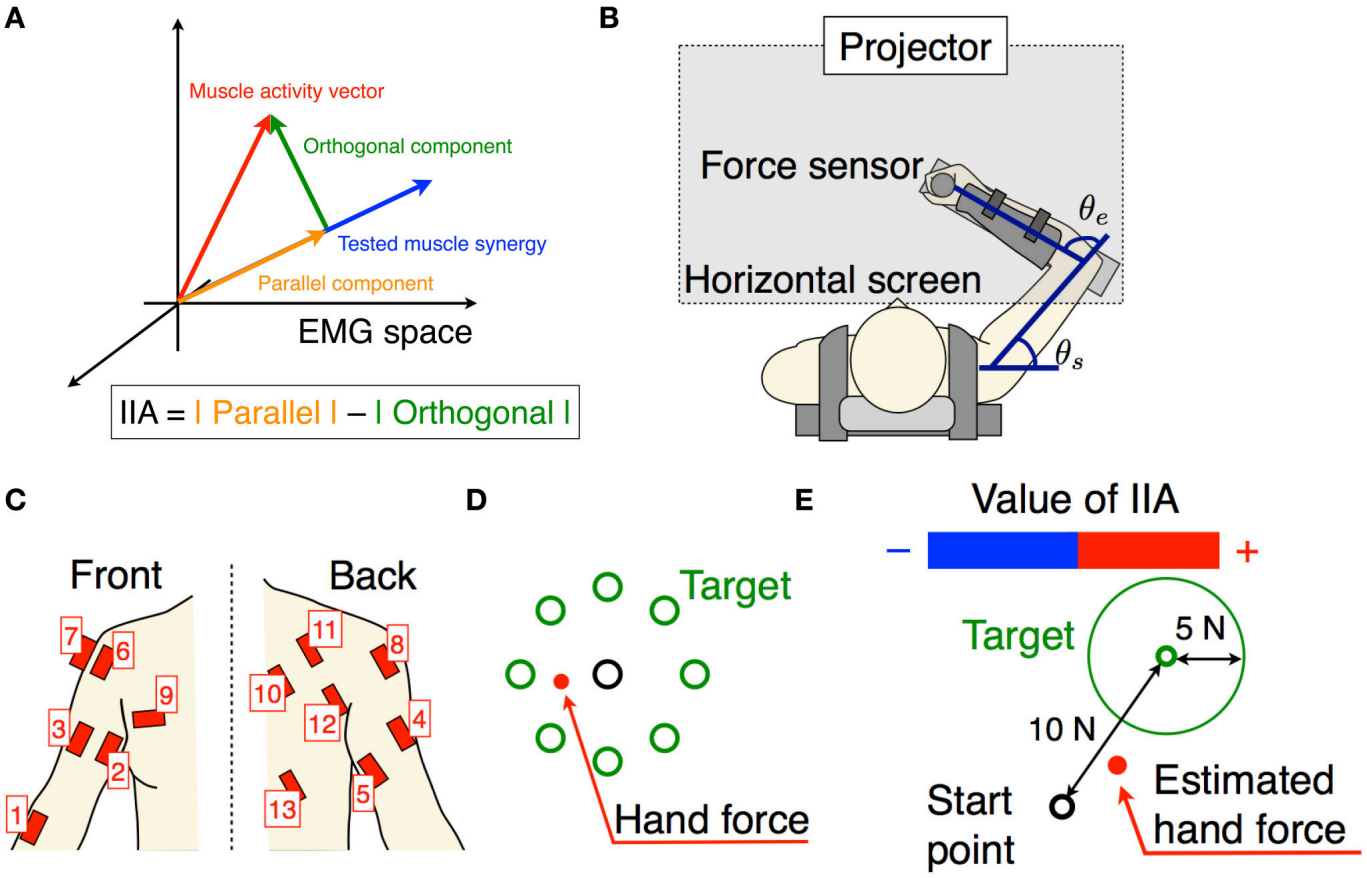
Experimental setup. **(A)** The schematic of the tested muscle synergy, current muscle activity of subject, and parallel and orthogonal components in EMG space. The IIA criterion is defined by a difference between the parallel and orthogonal components. **(B)** Subjects are seated on a chair, wearing a seat belt to fix their trunks, and holding a force sensor with their right hand. The θ_*e*_ and θ_*s*_ denote elbow and shoulder angles, respectively. Wrist movements are restricted by a plastic cuff. The horizontal screen showed the information for the task. **(C)** Thirteen muscle activities related to the shoulder and elbow movements were measured. **(D)** The information shown for the isometric force production task. Eight directional targets and actual (measured) hand force or estimated hand force are shown by circles. **(E)** The information shown for the voluntary activation task. The target point, target area, and estimated hand force are shown by circles. The positive negative values of the IIA correspond to the lengths of the red and blue bars, respectively.

In this study, we tested the hypothesis that the muscle synergies extracted by a conventional method are voluntarily activatable by neurophysiological processes. We extracted time-invariant muscle synergies with the non-negative matrix factorization (NMF) method (Lee and Seung, 1999) from muscle activities during isometric force production tasks. We varied the number of muscle synergies used in the NMF method. According to previous studies (Torres-Oviedo et al., 2006; d'Avella et al., 2008; Berger et al., 2013; Borzelli et al., 2013; de Rugy et al., 2013; Gentner et al., 2013; Russo et al., 2014), conventional muscle synergies are defined by the number of muscle synergies *N* that yield over 90% variability accounted for (VAF, *N*_*VAF*_) or coefficient of determination (CD, *N*_*CD*_). Moreover, to consider a muscle synergy with higher reconstruction ability, an additional muscle synergy model with *N*_*AD*_ = *N*_*VAF*_+ 1 or *N*_*AD*_ = *N*_*CD*_ + 1 (whichever was larger) was evaluated. We tested whether the individual conventional muscle synergies could be voluntarily activated or not. According to our assumptions, by evaluating the muscle synergies estimated by *N*_*AD*_, we could investigate either if subjects could voluntarily activate only muscle synergies estimated from *N*_*VAF*_ and *N*_*CD*_ or if subjects could also robustly activate the additional muscle synergies. Moreover, we evaluated the additional muscle synergies to consider the variation in the number of muscle synergies due to the threshold condition in the conventional synergy extraction method. We also tested voluntary activation of the extended muscle synergies (muscle synergy model with *N*_*EX*_ = *N*_*AD*_ + 1, 2…) that showed low similarity (inner product of vectors) to the conventional muscle synergies and each other. To evaluate voluntary activation of the muscle synergies, we defined an “index of independent activation” that reflects similarities between the tested single muscle synergy vector and the muscle activation vector of a subject in EMG space (Figure 1A). Subjects had visual feedback of the index in real time. They were instructed to produce a muscle activity pattern similar to the tested muscle synergy vector. We evaluated the index of independent activations and evaluated the voluntary activation of each muscle synergy.

## Materials and methods

### Subjects

Ten healthy right-handed male volunteers participated in our experiments. Their average age was 24.9 (range: 21–34 years). The experiments were approved by the ethics committee at Advanced Telecommunication Research Institute International. All subjects received explanations about the experimental procedure and gave their written informed consent.

### Apparatus

We used a twin visuomotor and haptic interface system (TVINS) to record the hand force at 2,000 Hz (Figure 1B). The arm of the TVINS was fixed so as not to move the subject's hand during experiments. Subjects sat on an adjustable chair while grasping the handle of the TVINS. The subject's hand (the handle of the TVINS) was adjusted so that the elbow angle θ_*e*_ was 90° and the shoulder angle θ_*s*_ was 45°. The subject's forearm was secured to a support beam on the horizontal plane by a plastic cuff. The plastic cuff also restricted the subject's wrist motion. The hand force of the subject was measured by the load cell attached on the TVINS arm. EMG was also recorded from 13 muscles related to elbow and shoulder movements at 2000 Hz (1, brachioradialis; 2, biceps brachii short head; 3, biceps brachii long head; 4, triceps brachii lateral head; 5, triceps brachii long head; 6, anterior deltoid; 7, middle deltoid; 8, posterior deltoid; 9, pectoralis major; 10, middle trapezius; 11, infraspinatus; 12, teres major; 13, latissimus dorsi) as shown in Figure 1C. Muscles used in the measurements were determined based on an earlier study of muscle synergy in an isometric force production task (Berger et al., 2013). A projector displayed task information on a horizontal screen that was placed above the subject's arm. The screen prevented the subjects from directly seeing their arm.

### Task procedures

First, subjects performed a simple isometric force production task using their actual hand force. We extracted muscle synergies and estimated an EMG-to-force mapping matrix from the hand force and EMG data measured during the isometric force production task. Using the EMG-to-force mapping matrix, we calculated the target force corresponding to the activation of one tested muscle synergy vector (the muscle synergy vector from EMG space was projected onto the target hand force in the workspace). Next, subjects performed the isometric force production task using hand force estimated from their EMG information, and this was used to evaluate the EMG-to-force mapping matrix. Finally, they performed a voluntary activation task. Specifically, the above procedures were conducted step-by-step as follows:

1. Isometric force production by actual force task: Subjects produced 8 directional isometric hand forces using their right hand, as shown in Figure 1B. The magnitudes of the isometric forces were 10, 15, and 20 N. Subjects continued to produce the force for 2 s per each directional force. The screen showed the 8 directional targets and the subject's current actual hand force (Figure 1D). Subjects performed the task with the same force target twice.
2. Estimation of EMG-to-force mapping matrix and extraction of muscle synergies: The data from hand force and EMG measured during the isometric force production task were used to estimate an EMG-to-force mapping matrix and extract muscle synergies. Using the hand force ***F***_*r*_ and EMG pattern ***m***_*r*_ during the rise of the hand force, the EMG-to-force mapping matrix ***H*** was estimated by the least squares method (***F***_*r*_ = ***Hm***_*r*_). We calculated the CD of force reconstruction with training data (***F***_*r*_ and ***m***_*r*_).

Next, we extracted muscle synergies from the EMG patterns ***m***_*r*_. We considered a time-invariant muscle synergy model (spatial pattern of muscle coordination):

(1) $$\begin{array}{r} {\text{m}}_{r}\left(t\right)=\sum_{i=1}^{N}c_{i}\left(t\right){\text{w}}_{\text{i}}+\varepsilon , \end{array}$$

where ***w***_*i*_ was the muscle synergy vector, *c*_*i*_ was the coefficient of activation, ε was the residual, and *N* was the variable number of muscle synergy vectors. Using the NMF method, ***w***_*i*_ and *c*_*i*_ were estimated. We performed the NMF algorithm 10 times to avoid a local solution, and adopted the results that showed the highest value of the CD. We changed the number of muscle synergies from 1 to 13 (the number of measured muscles), and extracted all 13 kinds of muscle synergy sets. In many cases, the muscle synergy in a motor task was extracted by the number of muscle synergies *N* that represented the minimum value over a threshold of the index of reconstruction (VAF or CD). Then, we calculated the index of reconstruction of the original EMG pattern for each muscle synergy, i.e., the VAF and CD were calculated as follows:

(2) $$\begin{array}{r} VAF=1-\frac{\Sigma_{n}\Sigma_{t}{\left(m-cw\right)}^{2}}{\Sigma_{n}\Sigma_{t}m}\\ CD=1-\frac{\Sigma_{n}\Sigma_{t}{\left(m-cw\right)}^{2}}{\Sigma_{n}\Sigma_{t}{\left(m-\bar{m}\right)}^{2}}, \end{array}$$

where *n* was the number of muscles, *t* was the time, and $\bar{m}$ was the mean value of *m*. The symbol ΣΣ denotes the double sum over all elements in the matrices. Using these indices of reconstruction, we defined the conventional muscle synergies and the extended muscle synergies. According to the NMF method, when a larger value for the number of muscle synergies *N* is used, a sparser muscle synergy is extracted. Hence, muscle synergies with differing sparsities can be extracted depending on the value chosen for the number of muscle synergies *N*. The conventional muscle synergies were extracted by the number of muscle synergies *N*_*VAF*_ and *N*_*CD*_, which were the minimum numbers corresponding to VAF ≥ 0.9 and CD ≥ 0.9, respectively. Moreover, the conventional muscle synergies included the additional muscle synergies extracted by *N*_*AD*_, which was one larger than *N*_*VAF*_ or *N*_*CD*_ (whichever was larger). Thus, the conventional muscle synergies included three types of muscle synergies extracted by *N* = *N*_*VAF*_, *N*_*CD*_, and *N*_*AD*_. The muscle synergies extracted by *N*_*EX*_ > *N*_*AD*_ were defined as the extended muscle synergies.

**Figure 2:**
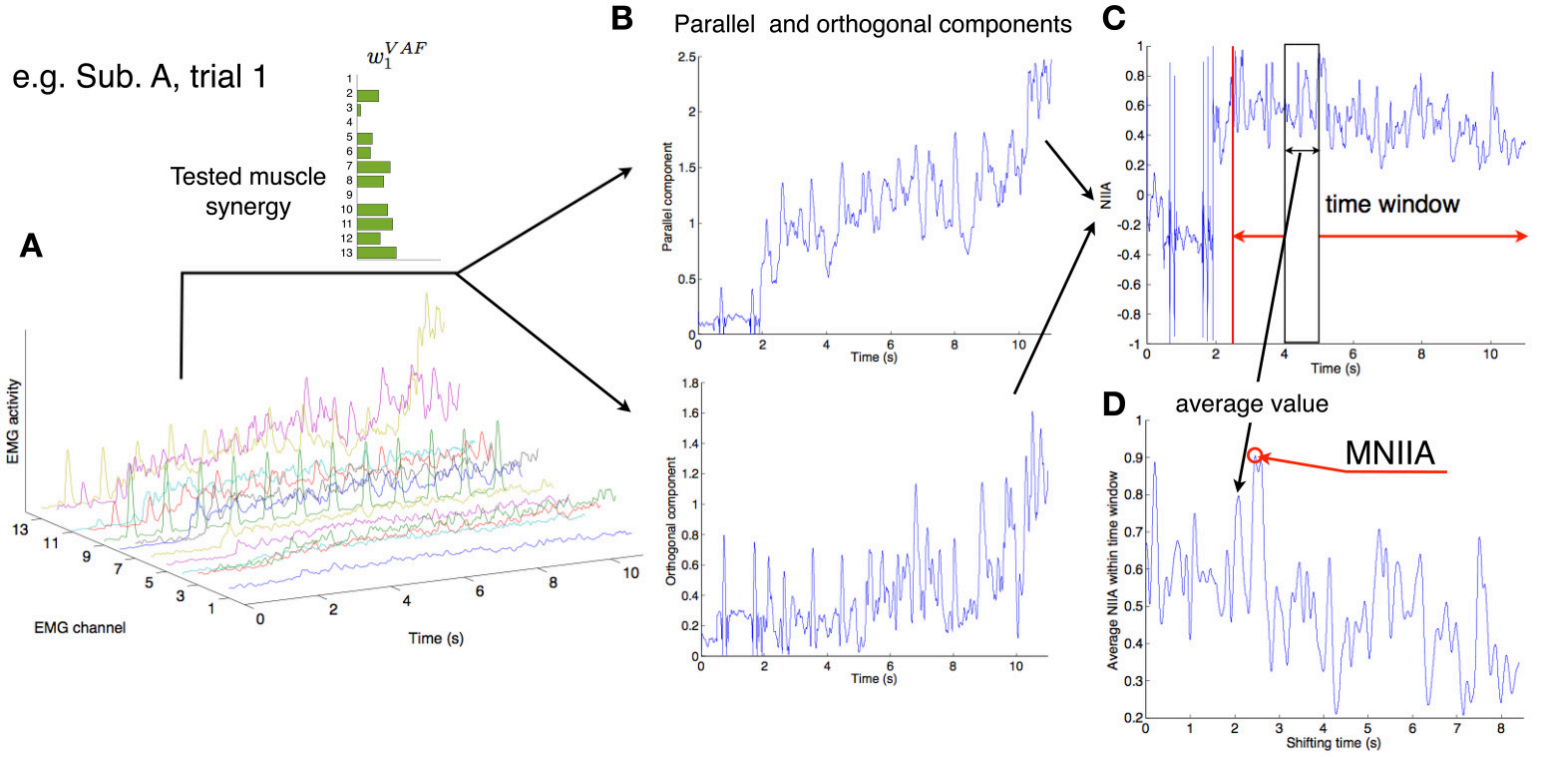
Calculation of MNIIA criterion. **(A)** The 13 EMG activity patterns of Subject A in trial 1. In trial 1, the tested muscle synergy vector was *w*_1_^*VAF*^. **(B)** The 13 EMG activities were transformed into the parallel and orthogonal components in real time (Figure 1A). The difference between the parallel and orthogonal components is the IIA criterion. The value of IIA is shown to the subjects in real time (Figure 1E). **(C)** In off-line analysis, the IIA is normalized (NIIA) and averaged within the time window. **(D)** The averaged NIIA within the time window is calculated at all time points during analysis duration (from 2.5 to 11 s after task onset). We determined a maximum NIIA (MNIIA) in each trial.

3. Isometric force production by estimated force task: Subjects produced 8 directional isometric hand forces using estimated hand force. The hand force ***F***_*e*_ was estimated using the EMG-to-force mapping matrix ***H*** and EMG ***m*** as follows: ***F***_*e*_ = ***Hm***. The magnitude of the target force was 10 N only. The screen showed the 8 directional targets and the estimated hand force instead of actual hand force. We recorded the hand force and EMG data as the test data and from it evaluated the CD of force reconstruction.
4. Voluntary activation task: We defined the voluntary activation of individual muscle synergies as producing an EMG pattern parallel to the single muscle synergy vector (Figure 1A). Therefore, subjects tried to produce EMG patterns parallel to the single tested muscle synergy vector. We tested all muscle synergy vectors of the conventional muscle synergies. We also tested the muscle synergy vectors in the extended muscle synergy group, which were not similar to the conventional muscle synergies and to each other (the inner product of two muscle synergy vectors was smaller than 0.9). We adjusted the total number of tested muscle synergy vectors to be no larger than 30 to avoid testing overlapping extended muscle synergies and to reduce the subject's fatigue. To assist in the voluntary activation of the tested muscle synergy, we defined an index of independent activation (IIA) as follows:
  (3) $$\begin{array}{r} IIA\left(m\right)=||\left(m^{T}{\text{w}}_{test}\right){\text{w}}_{test}||-||m-\left(m^{T}{\text{w}}_{test}\right){\text{w}}_{test}||, \end{array}$$
  where ***w***_*test*_ was the tested muscle synergy vector. The two components correspond to the parallel and orthogonal components of EMG, respectively (Figures 1A, 2A,B). The IIA is consistent with the independent activatability of muscle synergies. A positive IIA indicates that the parallel component is larger than the orthogonal component. Subjects observed the visual feedback of the IIA in real time, and attempted to match their voluntary activation to the tested muscle synergy.

In one trial, subjects tried to produce EMG patterns parallel to the single tested muscle synergy for 11 s. They performed 5 trials for every tested muscle synergy vector. During the first second subjects were at rest, and the average EMG activity was calculated and used to remove the offset of the EMG data in every trial. During the subsequent 10 s, subjects produced hand forces and tried to voluntarily generate muscle activity patterns parallel to the single test muscle synergy vector by using the IIA and target force feedback. The screen showed the target force, target force area, estimated hand force, and IIA information (Figure 1E). The target force ***F***_*t*_ was calculated as follows: ***F***_*t*_ = *C****Hw***_*test*_. The target force direction was calculated by the term ***Hw***_*test*_, and the coefficient *C* was adjusted so that the target force magnitude was 10 N. The target area was defined as the area within a circle centered on ***F***_*t*_ having a radius of 5 N. A positive IIA was shown as a red bar and a negative one was shown as a blue bar, with the value of IIA corresponding to the length of the bar. Subjects were instructed to enlarge the red bar while moving the point corresponding to the estimated hand force within the target area instead of producing accurate target force. Moreover, they were told that changes to the bar corresponded to changes in the balance of muscle activation among parts of the body, and were instructed to heuristically find the combination of muscle activation that produced the largest red bar. Between trials, subjects took a short break when they felt fatigue.

### Data analysis

All recorded data in the isometric force production task were down-sampled with a 10-point average. EMG data were full-wave rectified, filtered using a second-order Butterworth low-pass filter with a 3-Hz cut-off frequency, and normalized to the highest value during the task with 10 N target. Force data were also filtered using a second-order Butterworth low-pass filter with a 3-Hz cut-off frequency. We defined a dynamic phase as the duration from start to finish of a change in hand force. Using EMG data ***m***_*r*_ and force data ***F***_*r*_ from the dynamic phases under all conditions of the task, we estimated the EMG-to-force mapping matrix ***H*** through the least squares method (***F***_*r*_ = ***Hm***_*r*_). We also used EMG data ***m***_*r*_ to extract muscle synergies through the NMF algorithm, which divided EMG data ***m***_*r*_ into the muscle synergies ***w*** and the coefficient of activation ***c*** (Equation 1). We determined *N*_*VAF*_, *N*_*CD*_, *N*_*AD*_, and *N*_*EX*_ according to the VAF and CD (Equation 2), and extracted the conventional muscle synergies and extended muscle synergies. In the voluntary activation task, real-time EMG data were full-wave rectified, filtered using a second-order Butterworth low-pass filter with a 3-Hz cut-off frequency, and normalized to the highest value of EMG in the isometric force production task with 10 N target. The IIA was calculated as Equation (3) and was visually feed-backed to subjects as shown in Figure 1E. In every trial, we removed the offset of the EMG data by normalizing to the initial 1 s of data taken at rest.

To evaluate voluntary activation of each muscle synergy, we normalized IIA. We calculated normalized IIA (NIIA) as follows:

(4) $$\begin{array}{r} NIIA\left(m\right)=\frac{IIA}{||\left(m^{T}w_{test}\right)w_{test}||+||m-\left(m^{T}w_{test}\right)w_{test}||.} \end{array}$$

We shifted the 100 ms time window from 2.5 to 11 s after trial onset, and calculated the mean value of NIIA in the time window at each step (Figure 2C). We adopted the maximum value of the NIIA (MNIIA, Figure 2D), and compared it to the MNIIA of tasks with different targets (and with different tested muscle synergies). When we used a relatively wide time window, i.e., 1 s, we saw the same results.

Using the MNIIA, we first evaluated voluntary activation of the conventional muscle synergies by two criteria: (1) the MNIIA in the task with the target associated with the conventional muscle synergies was larger than 0, which means the parallel component was larger than the orthogonal component; and (2) the MNIIA was larger than the index of random activation, which was defined as *NIIA*(***m***_*rand*_). Each component of ***m***_*rand*_ was randomly generated (from 0 to 1) and normalized to a norm of 1. We used 10,000 ***m***_*rand*_ vectors and calculated the average value of the *NIIA*(***m***_*rand*_). We statistically evaluated the above two criteria by one-sample *t*-tests with Bonferroni corrections (α = 0.025). Second, we evaluated voluntary activation of the extended muscle synergies. To evaluate that the voluntary activation of the extended muscle synergies cannot be represented by the combination of conventional muscle synergies, and the degree of voluntary activation of the extended muscle synergies was the same as or larger than that of the conventional muscle synergies, we used the following two criteria: (1) the MNIIA in the task with the target associated with the extended muscle synergy was larger than the index of optimal combination of the conventional muscle synergies; and (2) the MNIIA was the same or larger than the average MNIIA of the conventional muscle synergies. The index of optimal combination of conventional muscle synergies was defined as *NIIA*(***m***_*opt*_), where ***m***_*opt*_ was the muscle activation matrix reconstructed by optimal combination of conventional muscle synergy ***w***_*conv*_ and optimal coefficient of activation *c*_*opt*_ as follows:

(5) $$\begin{array}{r} {\text{m}}_{opt}=\sum_{i=1}^{N}c_{opt}{\text{w}}_{conv.} \end{array}$$

The optimal coefficient of activation *c*_*opt*_ was defined by a solution of the following constrained optimization problem:

(6) $$\begin{array}{r} \text{min}.||cw_{conv}-w_{test}||\\ \text{ s}.\text{t}.\;c\geq 0 \end{array}$$

We used the nonnegative linear least-squares problem solver (*lsqnonneg* function) in MATLAB to solve the optimization problem. We used three types of conventional muscle synergies that were extracted by *N*_*VAF*_, *N*_*CD*_, and *N*_*AD*_. We statistically evaluated the above two criteria by one-sample *t*-tests with Bonferroni corrections (α = 0.025). Using these statistically evaluated results, we calculated the number of voluntarily activatable muscle synergies.

## Results

We show the number of conventional muscle synergies and the CD of force reconstruction of all subjects in Table 1. In nine out of ten subjects showed that the number of muscle synergies determined by the VAF was smaller than that determined by the CD. The only exception was Subject C, which showed the same number for the VAF and CD. These results indicate that using the VAF criterion, a smaller number of muscle synergies is extracted than when using the CD. The use of the CD of force reconstruction on training data showed a high value similar to that reported in an earlier study, i.e., 0.81 ± 0.05 in Berger et al. (2013). Moreover, the CD of the hand force reconstruction (0.811 ± 0.06) on the training data was not significantly different from the CD (0.783 ± 0.07) on the test data [a paired *t*-test; *p*-value: *p* = 0.273, *t*-value: *t*_(9)_ = 1.17, effect size: *r* = 0.36]. These results indicate that the hand force was well reconstructed from the EMG data.

**Table 1:** Results of muscle synergy extraction.

**Subject ID**	***N_*VAF*_***	***N_*CD*_***	***N_*AD*_***	**CD of force reconstruction with training data**	**CD of force reconstruction with test data**
A	4	5	6	0.727	0.788
B	3	4	5	0.814	0.690
C	3	3	4	0.877	0.805
D	2	3	4	0.836	0.832
E	4	5	6	0.723	0.818
F	4	5	6	0.839	0.849
G	3	5	6	0.767	0.662
H	4	5	6	0.795	0.768
I	4	5	6	0.875	0.885
J	4	5	6	0.862	0.732
Average ± SD	3.50 ± 0.71	4.50 ± 0.85	5.50 ± 0.85	0.811 ± 0.06	0.783 ± 0.07

*The N_VAF_ and N_CD_ denote the number of muscle synergies extracted by VAF and CD criteria, respectively. The N_AD_ denotes the number of muscle synergies that is one more than N_CD_ or N_VAF_(the larger one). The CD of force reconstruction is calculated by using the training data (EMG and force data in the isometric force production by actual force task) and the test data (EMG and force data in the isometric force production by estimated force task)*.

**Figure 3:**
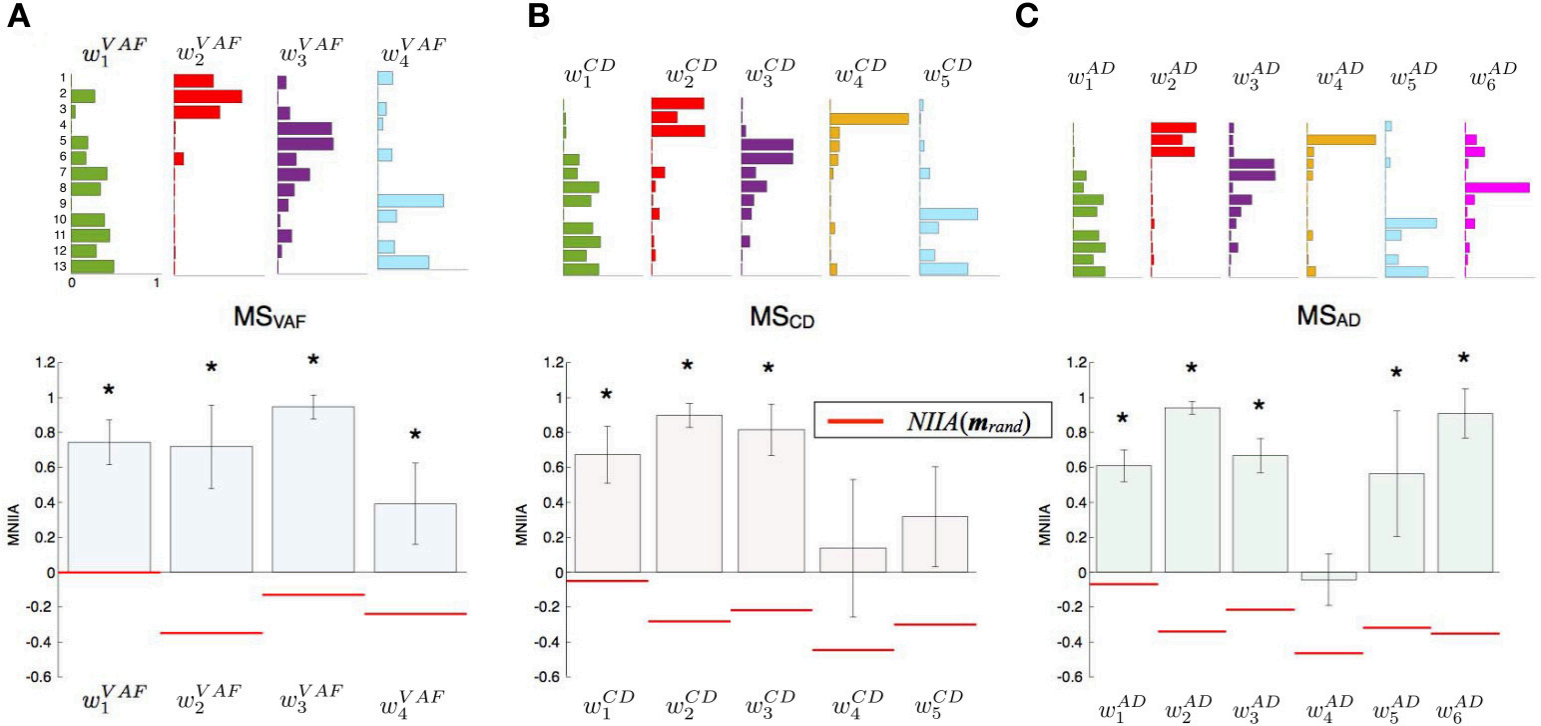
Evaluation of voluntary activation of conventional muscle synergies of a typical subject. Upper panels show muscle synergies extracted by *N*_*VAF*_**(A)**, *N*_*CD*_**(B)**, and *N*_*AD*_**(C)**. Lower panels show the MNIIA of each tested muscle synergy. MNIIA is the maximum normalized IIA in one trial (Figure 2), and its value denotes parallelism between the muscle activity of the subject and the tested muscle synergy (Figure 1A). Horizontal red bars denote the values of the index of random activation *NIIA*(***m***_*rand*_). The asterisk indicates that the following two criteria are satisfied: (1) the MNIIA is significantly larger than 0 (*p* < 0.025), and (2) the MNIIA is significantly larger than the *NIIA*(***m***_*rand*_) (*p* < 0.025).

Figure 3 shows the evaluation of voluntary activation of the conventional muscle synergies of a typical subject (Subject A). The top panels show the extracted muscle synergies. When the number of muscle synergies increased from *N*_*VAF*_ to *N*_*AD*_, the same colored muscle synergy vectors tended to be preserved (green, red, purple, and light blue), and the muscle synergy vectors with new weighting patterns (*w*_4_^*CD*^ and *w*_6_^*AD*^) were extracted. The lower figures show the MNIIA of all conventional muscle synergies. We evaluated data for the following two criteria: (1) the MNIIA is significantly larger than 0, and (2) the MNIIA is significantly larger than the *NIIA*(***m***_*rand*_). All *NIIA*(***m***_*rand*_), denoted by horizontal red lines, were smaller than 0. The asterisk indicates that both two criteria were satisfied. Details of statistical results are shown in Table S1 in Supplementary Materials. Subject A could voluntarily activate all muscle synergy vectors extracted by *N*_*VAF*_ [Figure 3A, *p* = 4.04 × 10^−6^–0.0196, *t*_(4)_ = 3.77–34.86, *r* = 0.88–1.00], but could not voluntarily activate the other conventional muscle synergies.

**Figure 4:**
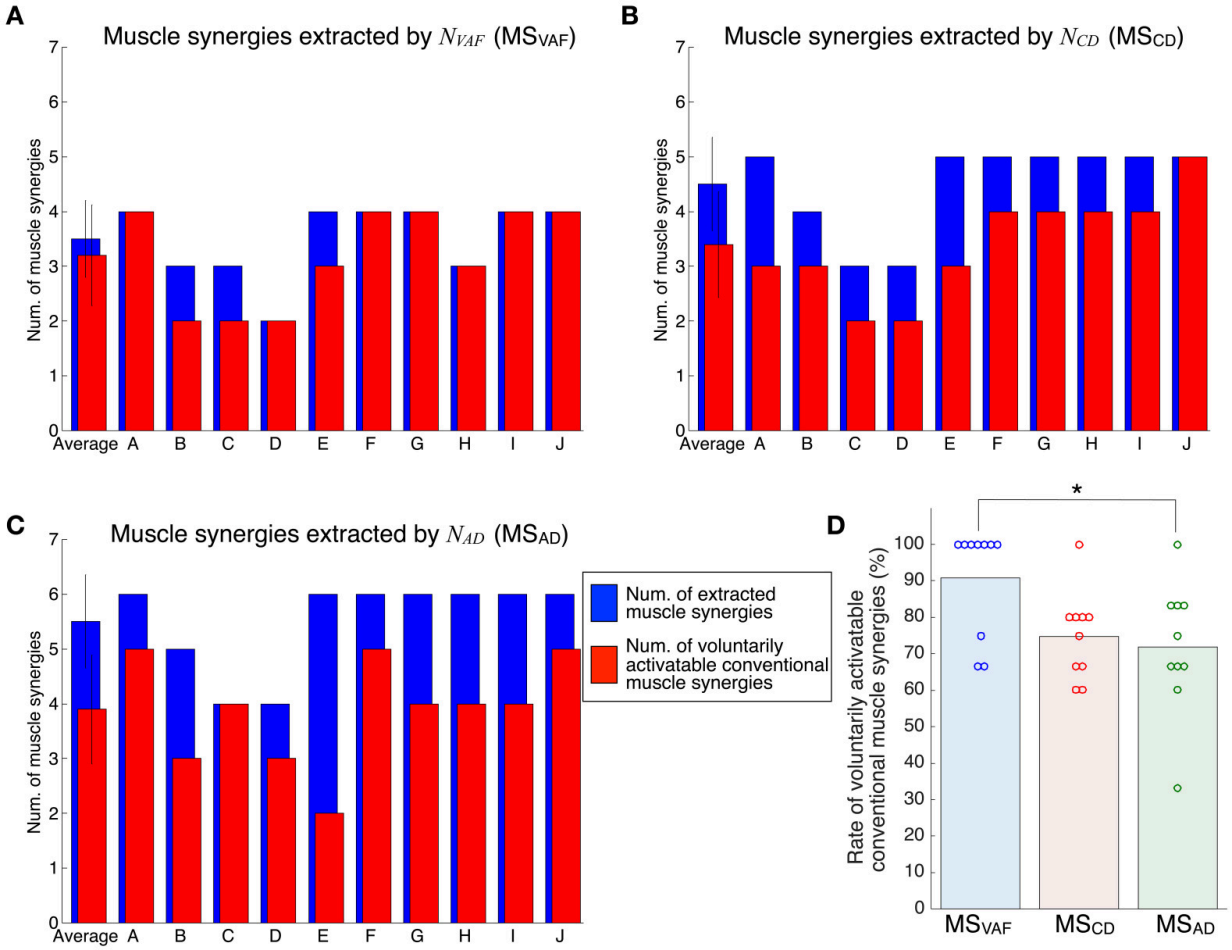
Evaluation of voluntary activation of conventional muscle synergies of all subjects. The number of extracted and voluntarily activatable conventional muscle synergies according to *N*_*VAF*_**(A)**, *N*_*CD*_**(B)**, and *N*_*AD*_**(C)**. **(D)** Rate of voluntarily activatable conventional muscle synergies. The bars and circles denote mean values and values of individual subjects, respectively. The asterisk indicates a significant difference (*p* < 0.05).

Figure 4 shows the evaluation of voluntary activation of the conventional muscle synergies of all subjects. Details of statistical results are shown in Table S1 in Supplementary Materials. Subjects could voluntarily activate an average of 90.8, 74.8, and 71.8% of the conventional muscle synergies extracted by *N*_*VAF*_, *N*_*CD*_, and *N*_*AD*_, respectively. A one-way ANOVA showed a significant effect of the type of conventional muscle synergy [*p* = 0.02, *F*-value: *F*_(2, 27)_ = 4.53, effect size: η^2^ = 0.25]. The *post-hoc* test with the Tukey-Kramer method showed that the rate of voluntarily activatable muscle synergies extracted by the *N*_*VAF*_ was significantly larger than that by the *N*_*AD*_. Moreover, the number of subjects who could voluntarily activate 100% of the conventional muscle synergies was the highest in the muscle synergy extracted by *N*_*VAF*_ (*n* = 7 for *N*_*VAF*_ and *n* = 1 for *N*_*CD*_ and *N*_*AD*_). When the number of muscle synergies increased from *N*_*VAF*_ to *N*_*AD*_, the rate of voluntarily activatable conventional muscle synergies and the number of subjects who could voluntarily activate 100% of the muscle synergies decreased. These results indicate that VAF can extract muscle synergies that can be voluntarily activated, i.e., independent muscle synergies. Moreover, the largest number of muscle synergies that could be voluntarily activated 100% of the extracted muscle synergies was 4 (*n* = 7).

**Figure 5:**
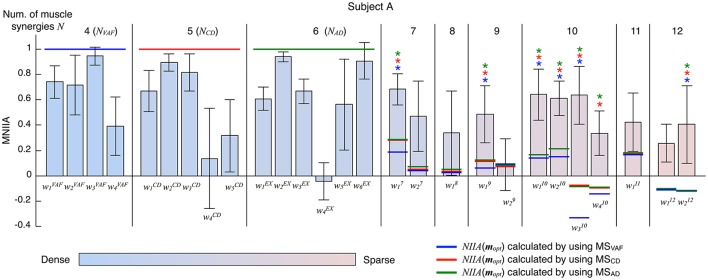
Evaluation of voluntary activation of extended muscle synergies of a typical subject. The bars and the error bars denote the mean values and the standard deviations of the MNIIA criterion across 5 trials. The vertical black lines separate muscle synergy groups according to the number of muscle synergies *N*. The *N*_*VAF*_, *N*_*CD*_, and *N*_*AD*_ of subject A are 4, 5, and 6, respectively. The data for the MNIIA of conventional muscle synergies are the same as data shown in Figure 3. The number of presented MNIIA results (vertical bars) is different from the number of muscle synergies *N*, because the tested muscle synergies were only the muscle synergy vectors that showed low similarity to the conventional muscle synergies and each other. The color bar indicates sparsity of each muscle synergy. The blue, red, and green horizontal lines denote values of *NIIA*(***m***_*opt*_) calculated by using muscle synergies extracted by *N*_*VAF*_ (MS_*VAF*_), *N*_*CD*_ (MS_*CD*_), and *N*_*AD*_ (MS_*AD*_), respectively. The blue, red, and green asterisks indicate that the following two criteria are satisfied: (1) MNIIA is significantly larger than the *NIIA*(***m***_*opt*_) (*p* < 0.025); and (2) MNIIA is significantly larger than (*p* < 0.025) or not significantly different (*p* > 0.025) from the mean value of MNIIA of conventional muscle synergies.

Figure 5 shows the evaluation of voluntary activation of the extended muscle synergies of a typical subject (Subject A). The extended muscle synergies were extracted by the number of muscle synergies that were more than *N*_*AD*_, and those that were not similar to the conventional muscle synergies and other extended muscle synergies were tested. We evaluated the voluntary activation of the extended muscle synergies by the following two criteria: (1) MNIIA is significantly larger than the *NIIA*(***m***_*opt*_); and (2) MNIIA is significantly larger than or not significantly different from the mean value of the MNIIA of the conventional muscle synergies. The asterisk indicates that both two criteria were satisfied. Details of statistical results are shown in Table S2 in Supplementary Materials. Almost all *NIIA*(***m***_*opt*_) were similar across different indices of reconstruction. Some MNIIAs of the extended muscle synergies reflected a constraint of optimal combination of the conventional muscle synergies (e.g., $w_{2}^{9}$). However, some MNIIAs could not be explained by the optimal combination of the conventional muscle synergies (e.g., $w_{1}^{7}$, $w_{1}^{9}$…). Therefore, these results show that subjects could voluntarily activate some extended muscle synergies.

**Figure 6:**
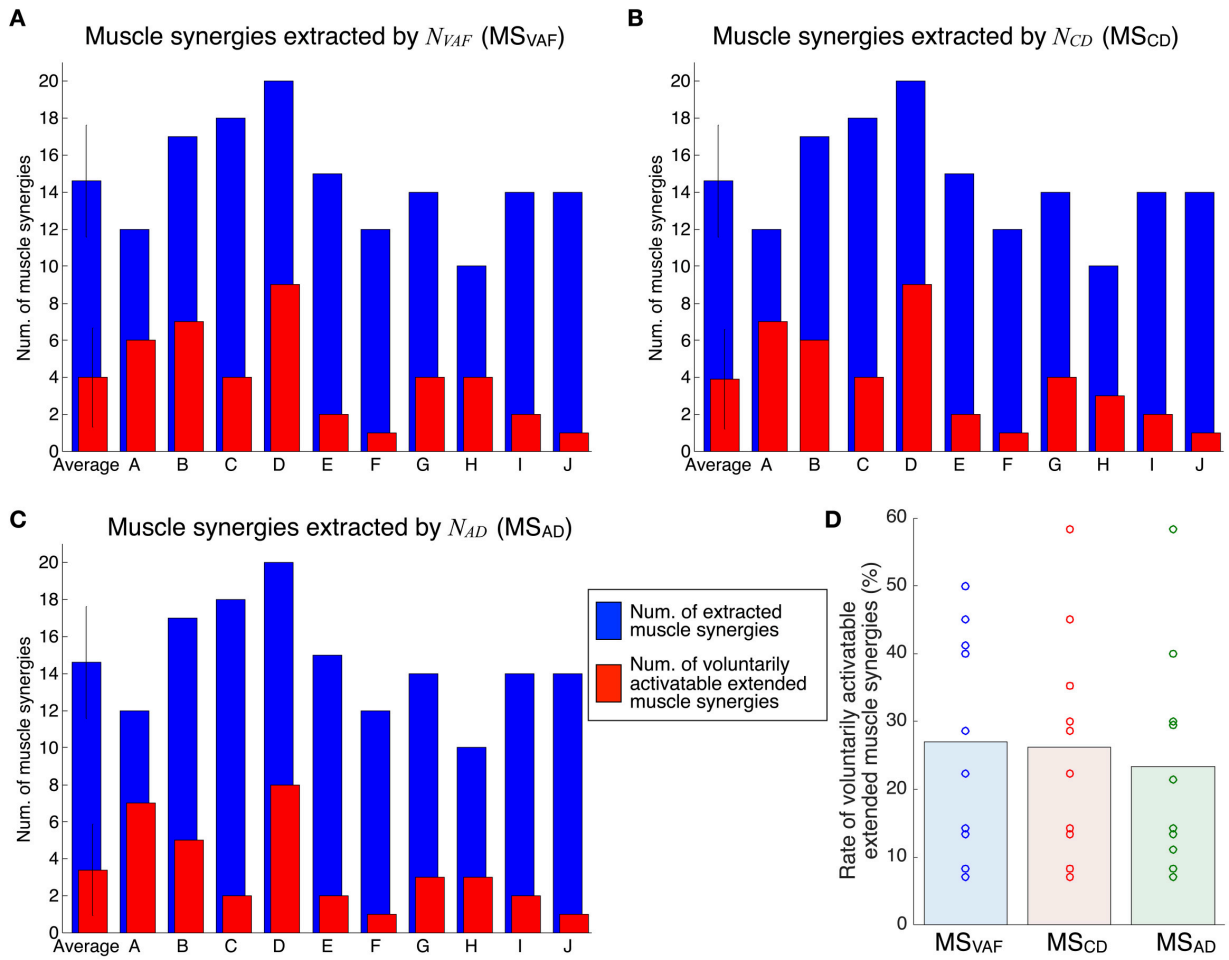
Evaluation of voluntary activation of extended muscle synergies of all subjects. The number of extracted and voluntarily activatable extended muscle synergies according to *N*_*VAF*_ **(A)**, *N*_*CD*_**(B)**, and *N*_*AD*_**(C)**. **(D)** Rate of voluntarily activatable extended muscle synergies. The bars and circles denote mean values and values of individual subjects, respectively.

Figure 6 shows the evaluation of voluntary activation of extended muscle synergies in all subjects. Details of statistical results are shown in Table S2 in Supplementary Materials. Subjects could voluntarily activate an average of 27.0, 26.3, and 23.3% of extended muscle synergies compared with *N*_*VAF*_, *N*_*CD*_, and *N*_*AD*_, respectively. The rates of voluntarily activatable extended muscle synergies were not significantly different between *N*_*VAF*_, *N*_*CD*_, and *N*_*AD*_ [*p* = 0.828, *F*_(2, 27)_ = 0.19, η^2^ = 0.0139]. These results indicate that an average of 25.5% of extended muscle synergies could be voluntarily activated, i.e., represented an independent module. In other words, an average of 74.5% of extended muscle synergies could not be voluntarily activated. This result also indicates that the muscle output pattern of subjects was restricted to a certain extent.

## Discussion

This study tested the hypothesis that the muscle synergies extracted by the conventional method are voluntarily and independently activatable by neurophysiological processes. We assumed that if the extracted individual muscle synergy could be voluntarily activated, the CNS contains neural circuits that can produce muscle activity patterns parallel to the tested muscle synergy vector, i.e., that represent individual muscle synergies. The neural circuits representing muscle synergies extracted by the NMF method can dominate common muscle activities. Because of the properties of the NMF method, the extracted factors are not necessarily orthogonal to each other. We extracted muscle synergies during the isometric force production task and then tested whether the subjects could voluntarily activate extracted individual muscle synergies under the same task conditions. We defined the index of independent activation as the difference between the parallel component and orthogonal component of tested muscle synergy vectors and muscle activity pattern vectors produced by the subjects (Figure 1A). Subjects visually checked the index in real time and tried to produce muscle activity patterns similar to the target muscle synergies. We defined voluntary activation of the conventional muscle synergies as satisfying the following two criteria: (1) the MNIIA was significantly larger than 0; and (2) the MNIIA was significantly larger than the index of random activation *NIIA*(***m***_*rand*_). As shown in Figure 4D, an average of 90.8% of the conventional muscle synergies extracted by *N*_*VAF*_ were voluntarily activatable, which indicates that almost all muscle synergies were voluntarily activatable. Figure 4D also showed that the lager the number of muscle synergies, e.g., extracted by *N*_*CD*_ or *N*_*AD*_, the smaller the rate of voluntarily activatable muscle synergies. Moreover, the number of subjects who could voluntarily activate 100% of the muscle synergies was largest for the muscle synergies extracted by *N*_*VAF*_ across three kinds of conventional muscle synergies. These results partially supported the hypothesis, and suggested that the conventional method could extract voluntarily activatable muscle synergies, i.e., independent muscle synergy groups, through the appropriate index of reconstruction. The Materials and Methods section in an earlier study by Torres-Oviedo et al. (2006) pointed out that the VAF is a more appropriate (strict) index of reconstruction than the CD. From our results showing that the VAF could extract muscle synergies that were 100% voluntarily activatable, this study experimentally supports their suggestion. Moreover, our results indicated that an inappropriate index of reconstruction extracts non-voluntarily activatable muscle synergies. In future work, we will examine whether these un-activatable muscle synergies are involuntarily activated muscle synergies or mathematical artifacts that are not from the CNS. In addition, we would be able to evaluate a capability of voluntary and independently activation of single muscle synergy with simulation technique, e.g., reinforcement learning of muscle synergies (Rueckert and d'Avella, 2013). We will conduct simulation experiments to approach the voluntary motor control via muscle synergies in future work.

In this study, we also examined voluntary activation of the extended muscle synergies. We defined the condition of the voluntary activation of the extended muscle synergies as a case in which the following two criteria were satisfied: (1) the MNIIA was significantly larger than the *NIIA*(***m***_*opt*_); and (2) the MNIIA was significantly larger than or not different from the mean MNIIA of the conventional muscle synergies. As shown in Figure 6D, an average of 25.5% of the extended muscle synergies could be voluntarily activated. This result indicates that the subjects could voluntarily activate the extended muscle synergies, which could not be explained by the optimal combination of the conventional muscle synergies. Our study experimentally showed that the CNS includes and is able to voluntarily use not only the conventional muscle synergies but also the sparse factor, i.e., the extended muscle synergies. There is a possibility that the conventional muscle synergies tested in this study showed activation as a result of chance synchronization of the sparse control modules. Our results experimentally indicated that there were some independent muscle synergy modules, but it remains unclear whether a relationship between the muscle synergy modules and the muscle activity primitives exist. We could test whether the extracted conventional muscle synergies are a superposition of the sparse muscle activity primitives in future work.

The simulation studies by De Groote et al. (2014) and Hirashima and Oya (2016) showed that the muscle synergy reflects a condition of the constraint of the task. According to their experimental results, the NMF algorithm could extract the muscle synergies from the model of muscle control without an assumption of the muscle synergy units. In this study, the largest number of muscle synergies of the conventional muscle synergy group that could be voluntarily activated 100% of extracted muscle synergies was 4, which was coincident with the task dimension (+*X*, −*X*, +*Y*, and −*Y*). However, the results of non-voluntarily activatable conventional muscle synergies and extended muscle synergies reflect the constraints of muscle activation patterns due to hierarchical control, which is more similar to the muscle synergy hypothesis than the optimal control of individual muscles (Fagg et al., 2002; Kurtzer et al., 2006; Tresch and Jarc, 2009). Earlier studies involving the modification of the spatial pattern of muscles in a virtual environment (virtual surgeries, Berger et al., 2013) and the modification of the output and degree of variability of one muscle in a virtual environment (de Rugy et al., 2012) also experimentally showed constraints of muscle activation patterns reflecting the hierarchical control similar to the muscle synergy. The results of the current and earlier studies suggest that multi-muscle control by the CNS is constrained by the hierarchical control of a small number of control modules. Moreover, our results showing the voluntary activation of extended muscle synergies suggest that the multi-muscle control system in the CNS could perform the task by using a finite number of control modules that are larger than the task dimensions and smaller than the individual muscles.

## Author contributions

ST conceived, designed, and performed the experiments; ST, HI analyzed the data and wrote the manuscript.

### Conflict of interest statement

ST and HI are affiliated with a commercial company, Advanced Telecommunications Research Institute International. The reviewer EC and handling Editor declared their shared affiliation.
