# Supplementary material for: Empirical Evaluation of Voluntarily Activatable Muscle Synergies
Source: Front Comput Neurosci. 2017 Sep 6;11:82. doi: 10.3389/fncom.2017.00082 (PMC5592215; doi:10.3389/fncom.2017.00082)
Supplement: Supplementary file 1 [file Table1.docx]

**Statistical tables**

**Table S1 | *p* values, *t* values and effect size associated with statistical evaluation of voluntary activation of the conventional muscle synergies.** The following two criteria were tested: 1) the MNIIA is significantly larger than 0; and 2) the MNIIA is significantly larger than *NIIA*(***m****_rand_*). The subscript ‘(s)’ means that the MNIIA of each conventional muscle synergy is significantly smaller than each criterion. The *w_i_^VAF^*, *w_i_^CD^*, and *w_i_^AD^* are conventional muscle synergies extracted by *N_VAF_*, *N_CD_*, and *N_AD_,* respectively. Gray filled data indicates that both criteria are satisfied according to a paired *t*-test with Bonferroni correction (*p* < 0.025).

| ID | | Sub. A | | Sub. B | | Sub. C | | Sub. D | |
| --- | --- | --- | --- | --- | --- | --- | --- | --- | --- |
| Criterion | | (1) | (2) | (1) | (2) | (1) | (2) | (1) | (2) |
| *w_1_^VAF^* | *p*  *t*_(4)_  *r* | 2.01×10^-4^  13.02  0.99 | 2.01×10^-4^  13.02  0.99 | 6.91×10^-5^  17.07  0.99 | 7.63×10^-5^  16.65  0.99 | 0.129  1.91  0.69 | 0.0083  4.84  0.92 | 0.0018  7.41  0.97 | 3.67×10^-4^  11.16  0.98 |
| *w_2_^VAF^* | *p*  *t*_(4)_  *r* | 0.0025  6.75  0.96 | 5.55×10^-4^  10.03  0.98 | 0.495  -0.75  0.35 | 0.248  1.35  0.56 | 0.0061  5.29  0.94 | 0.006  5.36  0.94 | 0.0037  6.10  0.95 | 0.0065  5.21  0.93 |
| *w_3_^VAF^* | *p*  *t*_(4)_  *r* | 6.78×10^-6^  30.61  1.00 | 4.04×10^-6^  34.86  1.00 | 0.005  5.59  0.94 | 0.002  6.99  0.96 | 2.84×10^-6^  38.09  1.00 | 1.07×10^-6^  48.57  1.00 | -  -  - | -  -  - |
| *w_4_^VAF^* | *p*  *t*_(4)_  *r* | 0.0196  3.77  0.88 | 0.0037  6.07  0.95 | -  -  - | -  -  - | -  -  - | -  -  - | -  -  - | -  -  - |
| *w_1_^CD^* | *p*  *t*_(4)_  *r* | 7.57×10^-4^  9.26  0.98 | 5.78×10^-4^  9.93  0.98 | 9.15×10^-4^  8.81  0.98 | 4.70×10^-4^  10.47  0.98 | 0.129  1.91  0.69 | 0.0083  4.84  0.92 | 5.74×10^-4^  9.94  0.98 | 9.85×10^-4^  15.60  0.99 |
| *w_2_^CD^* | *p*  *t*_(4)_  *r* | 7.65×10^-6^  29.70  1.00 | 2.56×10^-6^  39.08  1.00 | 0.0846  2.28  0.75 | 0.0102  4.58  0.92 | 0.0061  5.29  0.94 | 0.006  5.36  0.94 | 0.0085  4.82  0.92 | 0.0171  3.93  0.89 |
| *w_3_^CD^* | *p*  *t*_(4)_  *r* | 2.42×10^-4^  12.42  0.99 | 9.48×10^-5^  15.76  0.99 | 4.55×10^-4^  10.56  0.98 | 2.34×10^-4^  12.52  0.99 | 2.84×10^-6^  38.09  1.00 | 1.07×10^-6^  48.57  1.00 | 0.619  0.54  0.26 | 0.165  1.70  0.65 |
| *w_4_^CD^* | *p*  *t*_(4)_  *r* | 0.482  0.78  0.36 | 0.0298  3.30  0.86 | 5.37×10^-4^  10.12  0.98 | 2.18×10^-4^  12.75  0.99 | -  -  - | -  -  - | -  -  - | -  -  - |
| *w_5_^CD^* | *p*  *t*_(4)_  *r* | 0.0680  2.48  0.78 | 0.0085  4.82  0.92 | -  -  - | -  -  - | -  -  - | -  -  - | -  -  - | -  -  - |
| *w_1_^AD^* | *p*  *t*_(4)_  *r* | 1.13×10^-4^  15.07  0.99 | 7.42×10^-5^  16.76  0.99 | 1.88×10^-7^  75.09  1.00 | 7.84×10^-8^  93.14  1.00 | 0.023  3.59  0.87 | 0.0027  6.64  0.96 | 2.53×10^-4^  12.28  0.99 | 4.40×10^-5^  19.13  0.99 |
| *w_2_^AD^* | *p*  *t*_(4)_  *r* | 6.12×10^-7^  55.93  1.00 | 1.81×10^-7^  75.82  1.00 | 0.115  2.01  0.71 | 0.017  3.87  0.89 | 2.86×10^-5^  21.33  1.00 | 9.45×10^-6^  28.17  1.00 | 0.0051  5.57  0.94 | 0.0055  5.45  0.94 |
| *w_3_^AD^* | *p*  *t*_(4)_  *r* | 1.05×10^-4^  15.34  0.99 | 3.49×10^-5^  20.28  1.00 | 9.85×10^-5^  15.60  0.99 | 4.26×10^-5^  19.28  0.99 | 0.0076  4.98  0.93 | 0.0017  7.49  0.97 | 0.0012  8.13  0.97 | 3.35×10^-4^  11.43  0.99 |
| *w_4_^AD^* | *p*  *t*_(4)_  *r* | 0.549  -0.65  0.31 | 0.003  6.45  0.96 | 5.02×10^-4^  10.30  0.98 | 1.76×10^-4^  13.47  0.99 | 1.05×10^-5^  27.41  1.00 | 3.68×10^-6^  35.70  1.00 | 0.268  -1.29  0.54 | 0.345  1.07  0.47 |
| *w_5_^AD^* | *p*  *t*_(4)_  *r* | 0.0248  3.50  0.87 | 0.0054  5.48  0.94 | 0.0049_(s)_  -5.62  0.94 | 0.600  0.57  0.27 | -  -  - | -  -  - | -  -  - | -  -  - |
| *w_6_^AD^* | *p*  *t*_(4)_  *r* | 1.40×10^-4^  14.26  0.99 | 3.86×10^-5^  19.77  0.99 | -  -  - | -  -  - | -  -  - | -  -  - | -  -  - | -  -  - |

| ID | | Sub. E | | Sub. F | | Sub. G | | Sub. H | |
| --- | --- | --- | --- | --- | --- | --- | --- | --- | --- |
| Criterion | | (1) | (2) | (1) | (2) | (1) | (2) | (1) | (2) |
| *w_1_^VAF^* | *p*  *t*_(4)_  *r* | 0.0285  3.35  0.86 | 0.0097  4.65  0.92 | 0.0062  5.27  0.94 | 0.006  5.33  0.94 | 0.0023  6.90  0.96 | 0.0011  8.32  0.97 | 0.002  7.19  0.96 | 0.0017  7.47  0.97 |
| *w_2_^VAF^* | *p*  *t*_(4)_  *r* | 0.006  5.32  0.94 | 0.0017  7.44  0.97 | 0.0011  8.42  0.97 | 6.20×10^-4^  9.75  0.98 | 0.0016  7.58  0.97 | 9.23×10^-4^  8.79  0.98 | 8.81×10^-4^  8.90  0.98 | 3.74×10^-4^  11.11  0.98 |
| *w_3_^VAF^* | *p*  *t*_(4)_  *r* | 0.0061  5.29  0.94 | 0.0024  6.81  0.96 | 3.04×10^-4^  11.72  0.99 | 9.46×10^-5^  15.77  0.99 | 2.31×10^-6^  40.10  1.00 | 8.42×10^-7^  51.63  1.00 | 5.19×10^-5^  18.35  0.99 | 2.47×10^-5^  22.12  1.00 |
| *w_4_^VAF^* | *p*  *t*_(4)_  *r* | 5.57×10^-4^  10.02  0.98 | 1.51×10^-4^  14.00  0.99 | 2.02×10^-4^  13.00  0.99 | 4.80×10^-5^  18.71  0.99 | 9.08×10^-4^  8.83  0.98 | 3.60×10^-4^  11.21  0.98 | -  -  - | -  -  - |
| *w_1_^CD^* | *p*  *t*_(4)_  *r* | 0.0364  3.09  0.84 | 0.0079  4.93  0.93 | 0.0164  3.98  0.89 | 0.0148  4.10  0.90 | 0.0023  6.95  0.96 | 4.98×10^-4^  10.32  0.98 | 5.49×10^-4^  10.06  0.98 | 3.03×10^-4^  11.72  0.99 |
| *w_2_^CD^* | *p*  *t*_(4)_  *r* | 0.0099  4.61  0.92 | 0.002  7.15  0.96 | 9.07×10^-5^  15.93  0.99 | 4.83×10^-5^  18.69  0.99 | 1.93×10^-4^  13.15  0.99 | 6.16×10^-5^  17.57  0.99 | 8.36×10^-6^  29.05  1.00 | 3.51×10^-6^  36.12  1.00 |
| *w_3_^CD^* | *p*  *t*_(4)_  *r* | 7.39×10^-8^  94.92  1.00 | 2.63×10^-8^  122.88  1.00 | 8.35×10^-6^  29.06  1.00 | 2.13×10^-6^  40.94  1.00 | 8.57×10^-7^  51.41  1.00 | 2.43×10^-7^  70.49  1.00 | 0.0449  2.88  0.82 | 0.004  5.95  0.95 |
| *w_4_^CD^* | *p*  *t*_(4)_  *r* | 1.8×10^-4^  13.38  0.99 | 4.45×10^-5^  19.08  0.99 | 0.0035  6.17  0.95 | 6.85×10^-4^  9.50  0.98 | 0.0017  7.54  0.97 | 8.13×10^-4^  9.09  0.98 | 0.0226  3.61  0.87 | 0.004  5.94  0.95 |
| *w_5_^CD^* | *p*  *t*_(4)_  *r* | 0.001_(s)_  -8.52  0.97 | 0.601  -0.57  0.27 | 0.417  0.91  0.41 | 0.0751  2.39  0.77 | 0.0269  3.42  0.86 | 0.0027  6.64  0.96 | 0.0132  4.25  0.90 | 0.0056  5.42  0.94 |
| *w_1_^AD^* | *p*  *t*_(4)_  *r* | 0.0311  3.26  0.85 | 0.0075  4.99  0.93 | 0.0088  4.78  0.92 | 0.0058  5.38  0.94 | 0.0052  5.55  0.94 | 0.002  7.11  0.96 | 6.62×10^-10^  308.51  1.00 | 4.26×10^-10^  344.40  1.00 |
| *w_2_^AD^* | *p*  *t*_(4)_  *r* | 0.0345  3.15  0.84 | 0.0068  5.14  0.93 | 2.89×10^-4^  11.86  0.99 | 1.97×10^-4^  13.09  0.99 | 1.76×10^-7^  76.39  1.00 | 4.95×10^-8^  104.91  1.00 | 3.79×10^-4^  11.07  0.98 | 1.02×10^-4^  15.48  0.99 |
| *w_3_^AD^* | *p*  *t*_(4)_  *r* | 2.73×10^-5^  21.58  1.00 | 7.98×10^-6^  29.39  1.00 | 0.012  4.36  0.91 | 0.0029  6.48  0.96 | 0.0268  3.42  0.86 | 0.0113  4.45  0.91 | 0.051  2.76  0.81 | 0.0037  6.07  0.95 |
| *w_4_^AD^* | *p*  *t*_(4)_  *r* | 8.16×10^-4^  9.08  0.98 | 1.62×10^-4^  13.75  0.99 | 1.55×10^-4^  13.90  0.99 | 2.81×10^-5^  21.41  1.00 | 0.0117  4.39  0.91 | 0.0011  8.50  0.97 | 0.0018  7.33  0.96 | 4.37×10^-4^  10.67  0.98 |
| *w_5_^AD^* | *p*  *t*_(4)_  *r* | 0.0041_(s)_  -5.93  0.95 | 0.284  1.24  0.53 | 0.378  0.99  0.44 | 0.0462  2.85  0.82 | 0.0046  5.71  0.94 | 0.0014  7.95  0.97 | 5.91×10^-6^  31.69  1.00 | 1.18×10^-6^  47.43  1.00 |
| *w_6_^AD^* | *p*  *t*_(4)_  *r* | 0.160  -1.73  0.65 | 0.626  0.53  0.26 | 1.43×10^-5^  25.38  1.00 | 4.07×10^-6^  34.81  1.00 | 0.056  2.67  0.80 | 0.0051  5.58  0.94 | 0.685  0.44  0.21 | 0.0232  3.58  0.87 |

| ID | | Sub. I | | Sub. J | |
| --- | --- | --- | --- | --- | --- |
| Criterion | | (1) | (2) | (1) | (2) |
| *w_1_^VAF^* | *p*  *t*_(4)_  *r* | 1.12×10^-4^  15.11  0.99 | 7.29×10^-5^  16.84  0.99 | 5.87×10^-5^  17.79  0.99 | 3.95×10^-5^  19.65  0.99 |
| *w_2_^VAF^* | *p*  *t*_(4)_  *r* | 0.0026  6.70  0.96 | 0.0012  8.19  0.97 | 3.04×10^-4^  11.71  0.99 | 6.85×10^-5^  17.12  0.99 |
| *w_3_^VAF^* | *p*  *t*_(4)_  *r* | 0.0021  7.05  0.96 | 4.50×10^-4^  10.59  0.98 | 6.91×10^-5^  17.07  0.99 | 3.50×10^-5^  20.26  1.00 |
| *w_4_^VAF^* | *p*  *t*_(4)_  *r* | 7.15×10^-4^  9.39  0.98 | 2.72×10^-4^  12.05  0.99 | 6.60×10^-5^  17.27  0.99 | 1.02×10^-5^  27.51  1.00 |
| *w_1_^CD^* | *p*  *t*_(4)_  *r* | 9.69×10^-6^  28.00  1.00 | 3.05×10^-6^  37.40  1.00 | 0.0045  5.75  0.94 | 0.0016  7.61  0.97 |
| *w_2_^CD^* | *p*  *t*_(4)_  *r* | 0.113  2.02  0.71 | 0.0629  2.56  0.79 | 0.0012  8.26  0.97 | 4.77×10^-4^  10.43  0.98 |
| *w_3_^CD^* | *p*  *t*_(4)_  *r* | 0.0069  5.11  0.93 | 0.001  8.54  0.97 | 0.0016  7.63  0.97 | 2.51×10^-4^  12.30  0.99 |
| *w_4_^CD^* | *p*  *t*_(4)_  *r* | 0.0048  5.66  0.94 | 0.0012  8.20  0.97 | 1.28×10^-5^  26.11  1.00 | 4.55×10^-6^  33.83  1.00 |
| *w_5_^CD^* | *p*  *t*_(4)_  *r* | 0.0022  7.00  0.96 | 6.31×10^-4^  9.71  0.98 | 0.005  5.59  0.94 | 6.06×10^-4^  9.81  0.98 |
| *w_1_^AD^* | *p*  *t*_(4)_  *r* | 3.61×10^-5^  20.11  1.00 | 1.39×10^-5^  25.57  1.00 | 2.38×10^-4^  12.46  0.99 | 7.61×10^-5^  16.66  0.99 |
| *w_2_^AD^* | *p*  *t*_(4)_  *r* | 0.0071  5.08  0.93 | 0.0014  7.85  0.97 | 1.87×10^-4^  13.26  0.99 | 3.37×10^-5^  20.47  1.00 |
| *w_3_^AD^* | *p*  *t*_(4)_  *r* | 8.17×10^-4^  9.08  0.98 | 1.54×10^-4^  13.93  0.99 | 0.454  0.83  0.38 | 0.0057  5.39  0.94 |
| *w_4_^AD^* | *p*  *t*_(4)_  *r* | 0.444  0.85  0.39 | 0.109  2.05  0.72 | 0.006  5.32  0.94 | 8.46×10^-4^  9.00  0.98 |
| *w_5_^AD^* | *p*  *t*_(4)_  *r* | 0.0075  5.00  0.93 | 7.38×10^-4^  9.32  0.98 | 0.0113  4.45  0.91 | 0.0012  8.16  0.97 |
| *w_6_^AD^* | *p*  *t*_(4)_  *r* | 0.086  2.27  0.75 | 0.0106  4.53  0.91 | 3.31×10^-4^  11.46  0.99 | 1.28×10^-4^  14.60  0.99 |

**Table S2 | *p* values, *t* values and effect size associated with statistical evaluation of voluntary activation of extended muscle synergies.** The following two criteria were tested: 1) the MNIIA is significantly larger than *NIIA*(***m****_opt_*); and 2) the MNIIA is significantly larger than or significantly not different from mean value of MNIIA of conventional muscle synergies. The subscript ‘(s)’ means that the MNIIA of each extended muscle synergy is significantly smaller than each criterion. We used three types of conventional muscle synergies extracted by *N_VAF_* (a), *N_CD_* (b), and *N_AD_* (c). *MNIIA_VAF_*, *MNIIA_CD_*, and *MNIIA_AD_* are mean values of MNIIA associated with *N_VAF_*, *N_CD_*, and *N_AD_*, respectively. The *w_i_ ^j^* is extended muscle synergy, and subscript *j* is the number of muscle synergies. Gray filled data indicates that both criteria are satisfied according to a paired *t*-test with Bonferroni correction (*p* < 0.025).

(a)

| ID | | Sub. A | | Sub. B | | Sub. C | | Sub. D | |
| --- | --- | --- | --- | --- | --- | --- | --- | --- | --- |
| *MNIIA*_VAF_ | | 0.699 | | 0.312 | | 0.476 | | 0.647 | |
| Criterion | | (1) | (2) | (1) | (2) | (1) | (2) | (1) | (2) |
| *w_1_^5^* | *p*  *t*_(4)_  *r* | -  -  - | -  -  - | -  -  - | -  -  - | 0.0072  5.05  0.93 | 0.411  -0.92  0.42 | 0.0027  6.60  0.96 | 0.0067_(s)_  -5.15  0.93 |
| *w_2_^5^* | *p*  *t*_(4)_  *r* | -  -  - | -  -  - | -  -  - | -  -  - | -  -  - | -  -  - | 0.252  1.34  0.56 | 0.0077_(s)_  -4.96  0.93 |
| *w_1_^6^* | *p*  *t*_(4)_  *r* | -  -  - | -  -  - | 0.0886  2.24  0.75 | 0.144  1.82  0.67 | 0.0046  5.74  0.94 | 0.0635  -2.55  0.79 | 4.52×10^-4^  10.58  0.98 | 0.869  -0.18  0.087 |
| *w_1_^6^* | *p*  *t*_(4)_  *r* | -  -  - | -  -  - | 0.0021  7.11  0.96 | 0.0176_(s)_  3.90  0.89 | 0.0053  5.51  0.94 | 0.006_(s)_  -5.32  0.94 | 0.0021  7.09  0.96 | 1.45×10^-5^_(s)_  -25.31  1.00 |
| *w_1_^7^* | *p*  *t*_(4)_  *r* | 8.68×10^-4^  8.93  0.98 | 0.805  -0.26  0.13 | 0.0030  6.43  0.95 | 0.0029_(s)_  -6.51  0.96 | 0.0805  2.33  0.76 | 0.0261  -3.45  0.87 | 0.0034  6.24  0.95 | 0.0397  -3.01  0.83 |
| *w_2_^7^* | *p*  *t*_(4)_  *r* | 0.0259  3.45  0.87 | 0.138  -1.85  0.68 | 0.782  -0.30  0.15 | 6.07×10^-4^ _(s)_  -9.80  0.98 | -  -  - | -  -  - | -  -  - | -  -  - |
| *w_1_^8^* | *p*  *t*_(4)_  *r* | 0.102  2.12  0.73 | 0.0713  -2.44  0.77 | 0.0051  5.57  0.94 | 0.009  4.74  0.92 | 0.0107  4.51  0.91 | 1.53×10^-4^_(s)_  -13.95  0.99 | 0.1579  1.73  0.66 | 0.0361  -3.10  0.84 |
| *w_2_^8^* | *p*  *t*_(4)_  *r* | -  -  - | -  -  - | 0.0108  4.50  0.91 | 0.0667  -2.50  0.78 | -  -  - | -  -  - | 0.0868  2.26  0.75 | 0.0179_(s)_  -3.87  0.89 |
| *w_3_^8^* | *p*  *t*_(4)_  *r* | -  -  - | -  -  - | 0.0120  4.36  0.91 | 0.0485  2.81  0.81 | -  -  - | -  -  - | -  -  - | -  -  - |
| *w_4_^8^* | *p*  *t*_(4)_  *r* | -  -  - | -  -  - | 0.0369  -3.08  0.84 | 6.78×10^-4^_(s)_  -9.53  0.98 | -  -  - | -  -  - | -  -  - | -  -  - |
| *w_1_^9^* | *p*  *t*_(4)_  *r* | 0.0136  4.21  0.90 | 0.105  -2.09  0.72 | 0.318  1.14  0.50 | 0.0839  -2.29  0.75 | 0.0132  4.25  0.90 | 0.0046_(s)_  -5.73  0.94 | 0.0079  4.92  0.93 | 0.146  -1.80  0.67 |
| *w_2_^9^* | *p*  *t*_(4)_  *r* | 0.962  -0.05  0.03 | 0.0025_(s)_  -6.74  0.96 | 0.0023  6.94  0.96 | 0.002_(s)_  -7.21  0.96 | -  -  - | -  -  - | 0.0222  3.63  0.88 | 0.251  -1.34  0.56 |
| *w_3_^9^* | *p*  *t*_(4)_  *r* | -  -  - | -  -  - | -  -  - | -  -  - | -  -  - | -  -  - | 0.0625  2.56  0.79 | 0.0216_(s)_  -3.66  0.88 |
| *w_4_^9^* | *p*  *t*_(4)_  *r* | -  -  - | -  -  - | -  -  - | -  -  - | -  -  - | -  -  - | 0.895  0.14  0.07 | 0.0031_(s)_  -6.40  0.95 |
| *w_1_^10^* | *p*  *t*_(4)_  *r* | 0.0054  5.48  0.94 | 0.5584  -0.64  0.30 | 1.05×10^-4^  15.37  0.99 | 0.0037  6.08  0.95 | 7.57×10^-4^  9.26  0.98 | 0.193  1.56  0.62 | 0.0026  6.72  0.96 | 0.0691  -2.47  0.78 |
| *w_2_^10^* | *p*  *t*_(4)_  *r* | 0.0016  7.64  0.97 | 0.2209  -1.45  0.59 | 0.258  1.32  0.55 | 0.21  -1.49  0.60 | 0.826  0.23  0.12 | 0.0073_(s)_  -5.03  0.93 | 0.0047  5.68  0.94 | 0.0058_(s)_  -5.38  0.94 |
| *w_3_^10^* | *p*  *t*_(4)_  *r* | 6.73×10^-4^  9.55  0.98 | 0.5701  -0.62  0.30 | 0.0017  7.49  0.97 | 0.0722  2.43  0.77 | 0.0031  6.40  0.95 | 0.0067_(s)_  -5.17  0.93 | 0.0052  5.55  0.94 | 0.0058_(s)_  -5.37  0.94 |
| *w_4_^10^* | *p*  *t*_(4)_  *r* | 0.0037  6.07  0.95 | 0.0103_(s)_  -4.57  0.92 | -  -  - | -  -  - | -  -  - | -  -  - | 0.0756  -2.38  0.77 | 2.97×10^-5^_(s)_  -21.12  1.00 |
| *w_1_^11^* | *p*  *t*_(4)_  *r* | 0.0683  2.48  0.78 | 0.0548  -2.69  0.80 | 0.0621  2.57  0.79 | 0.832  0.23  0.11 | 1.83×10^-4^  13.32  0.99 | 0.0126_(s)_  -4.30  0.91 | 0.0082  4.88  0.93 | 0.0476  -2.82  0.82 |
| *w_2_^11^* | *p*  *t*_(4)_  *r* | -  -  - | -  -  - | 0.0012  8.19  0.97 | 0.439  -0.86  0.39 | -  -  - | -  -  - | 0.0038  6.02  0.95 | 0.244  -1.37  0.56 |
| *w_3_^11^* | *p*  *t*_(4)_  *r* | -  -  - | -  -  - | 2.59×10^-4^  12.20  0.99 | 0.0394  3.01  0.83 | -  -  - | -  -  - | 0.0014  7.86  0.97 | 6.14×10^-4^_(s)_  -9.77  0.98 |
| *w_4_^11^* | *p*  *t*_(4)_  *r* | -  -  - | -  -  - | -  -  - | -  -  - | -  -  - | -  -  - | 2.68×10^-4^  12.10  0.99 | 0.163  1.70  0.65 |
| *w_1_^12^* | *p*  *t*_(4)_  *r* | 0.0056  5.43  0.94 | 0.0028_(s)_  -6.53  0.96 | 0.0562  2.66  0.80 | 0.0109_(s)_  -4.49  0.91 | 0.231  1.41  0.58 | 0.0843  -2.29  0.75 | 2.41×10^-5^  22.26  1.00 | 0.388  0.97  0.44 |
| *w_2_^12^* | *p*  *t*_(4)_  *r* | 0.0195  3.78  0.88 | 0.0997  -2.13  0.73 | -  -  - | -  -  - | 0.026  3.45  0.87 | 0.0023_(s)_  -6.90  0.96 | -  -  - | -  -  - |
| *w_3_^12^* | *p*  *t*_(4)_  *r* | -  -  - | -  -  - | -  -  - | -  -  - | 0.0496  2.78  0.81 | 0.018_(s)_  -3.87  0.89 | -  -  - | -  -  - |
| *w_1_^13^* | *p*  *t*_(4)_  *r* | -  -  - | -  -  - | -  -  - | -  -  - | 0.0669  2.50  0.78 | 4.96×10^-4^_(s)_  -10.33  0.98 | -  -  - | -  -  - |
| *w_2_^13^* | *p*  *t*_(4)_  *r* | -  -  - | -  -  - | -  -  - | -  -  - | 0.513  -0.72  0.34 | 0.0065_(s)_  -5.20  0.93 | -  -  - | -  -  - |
| *w_3_^13^* | *p*  *t*_(4)_  *r* | -  -  - | -  -  - | -  -  - | -  -  - | 0.0025  6.73  0.96 | 0.0418  -2.95  0.83 | -  -  - | -  -  - |
| *w_4_^13^* | *p*  *t*_(4)_  *r* | -  -  - | -  -  - | -  -  - | -  -  - | 2.96×10^-5^  21.13  1.00 | 5.58×10^-5^_(s)_  -18.02  0.99 | -  -  - | -  -  - |
| *w_5_^13^* | *p*  *t*_(4)_  *r* | -  -  - | -  -  - | -  -  - | -  -  - | 4.36×10^-4^  10.67  0.98 | 0.0053_(s)_  -5.52  0.94 | -  -  - | -  -  - |

| ID | | Sub. E | | Sub. F | | Sub. G | | Sub. H | |
| --- | --- | --- | --- | --- | --- | --- | --- | --- | --- |
| *MNIIA*_VAF_ | | 0.609 | | 0.736 | | 0.764 | | 0.792 | |
| Criterion | | (1) | (2) | (1) | (2) | (1) | (2) | (1) | (2) |
| *w_1_^7^* | *p*  *t*_(4)_  *r* | 0.525  0.70  0.33 | 1.05×10^-4^_(s)_  -15.36  0.99 | 0.799  -0.27  0.13 | 0.0011_(s)_  -8.43  0.97 | 0.0159  4.01  0.90 | 0.269  -1.28  0.54 | 0.0025  6.74  0.96 | 0.0424  -2.94  0.83 |
| *w_2_^7^* | *p*  *t*_(4)_  *r* | -  -  - | -  -  - | -  -  - | -  -  - | 0.107  2.07  0.72 | 0.0712  -2.44  0.77 | 3.79×10^-4^  11.07  0.98 | 0.968  0.043  0.022 |
| *w_1_^8^* | *p*  *t*_(4)_  *r* | 0.0024  6.86  0.96 | 0.590  0.59  0.28 | 0.603  0.56  0.27 | 0.127  -1.93  0.69 | 0.393  -0.96  0.43 | 3.38×10^-4^_(s)_  -11.40  0.98 | 0.0527  2.73  0.81 | 1.79×10^-6^_(s)_  -42.78  1.00 |
| *w_2_^8^* | *p*  *t*_(4)_  *r* | -  -  - | -  -  - | 0.951  0.065  0.033 | 0.0044_(s)_  -5.81  0.95 | 0.0144  4.14  0.90 | 0.0119_(s)_  -4.37  0.91 | -  -  - | -  -  - |
| *w_3_^8^* | *p*  *t*_(4)_  *r* | -  -  - | -  -  - | 0.498  0.75  0.35 | 0.0214_(s)_  -3.67  0.88 | 0.0068  5.13  0.93 | 0.225  -1.43  0.58 | -  -  - | -  -  - |
| *w_1_^9^* | *p*  *t*_(4)_  *r* | 0.932  -0.09  0.045 | 0.0018_(s)_  -7.38  0.97 | 0.84  0.22  0.11 | 0.0341  -3.16  0.85 | 0.0049  5.64  0.94 | 0.0176_(s)_  -3.89  0.89 | 0.0711  2.44  0.77 | 0.0059_(s)_  -5.36  0.94 |
| *w_2_^9^* | *p*  *t*_(4)_  *r* | -  -  - | -  -  - | 0.289  1.22  0.52 | 0.0095_(s)_  -4.67  0.92 | 0.112  2.03  0.71 | 0.0049_(s)_  -5.63  0.94 | 0.0019  7.24  0.96 | 0.0027_(s)_  -6.60  0.96 |
| *w_3_^9^* | *p*  *t*_(4)_  *r* | -  -  - | -  -  - | 0.911  0.12  0.06 | 0.0079_(s)_  -4.93  0.93 | -  -  - | -  -  - | - | - |
| *w_4_^9^* | *p*  *t*_(4)_  *r* | -  -  - | -  -  - | 0.207  1.51  0.60 | 0.0015_(s)_  -7.71  0.97 | -  -  - | -  -  - | - | - |
| *w_1_^10^* | *p*  *t*_(4)_  *r* | 0.842  0.21  0.11 | 0.0033_(s)_  -6.29  0.95 | 0.472  0.79  0.37 | 0.0198_(s)_  -3.76  0.88 | 0.294  -1.21  0.52 | 0.0012_(s)_  -8.25  0.97 | 0.037  3.08  0.84 | 4.26×10^-5^_(s)_  -19.29  0.99 |
| *w_2_^10^* | *p*  *t*_(4)_  *r* | 0.180  1.62  0.63 | 0.376  -1.00  0.45 | 0.158  1.73  0.65 | 0.0025_(s)_  -6.74  0.96 | 0.121  1.96  0.70 | 0.0254  -3.48  0.87 | 0.006  5.33  0.94 | 0.330  -1.11  0.48 |
| *w_3_^10^* | *p*  *t*_(4)_  *r* | 0.529  -0.69  0.33 | 9.18×10^-4^_(s)_  -8.80  0.98 | -  -  - | -  -  - | -  -  - | -  -  - | -  -  - | -  -  - |
| *w_1_^11^* | *p*  *t*_(4)_  *r* | 0.137  -1.86  0.68 | 0.0018_(s)_  -7.40  0.97 | 0.0082  4.87  0.93 | 0.319  -1.14  0.49 | 0.950  -0.067  0.034 | 0.0164_(s)_  -3.98  0.89 | 0.0111  4.47  0.91 | 0.151  -1.77  0.66 |
| *w_2_^11^* | *p*  *t*_(4)_  *r* | 2.95×10^-5^  21.15  1.00 | 9.75×10^-6^_(s)_  -27.95  1.00 | 0.753  0.34  0.17 | 0.0293  -3.32  0.86 | 0.0067  5.16  0.93 | 0.0477  -2.82  0.82 | 0.154  1.76  0.66 | 0.0039_(s)_  -6.00  0.95 |
| *w_3_^11^* | *p*  *t*_(4)_  *r* | 0.0017  7.52  0.97 | 0.827  0.23  0.12 | -  -  - | -  -  - | -  -  - | -  -  - | 0.314  1.15  0.50 | 0.0013_(s)_  -8.10  0.97 |
| *w_1_^12^* | *p*  *t*_(4)_  *r* | -  -  - | -  -  - | -  -  - | -  -  - | 0.0033  6.25  0.95 | 0.0796  -2.34  0.76 | -  -  - | -  -  - |
| *w_2_^12^* | *p*  *t*_(4)_  *r* | -  -  - | -  -  - | -  -  - | -  -  - | 0.409  0.92  0.42 | 2.07×10^-6^_(s)_  -41.23  1.00 | -  -  - | -  -  - |
| *w_3_^12^* | *p*  *t*_(4)_  *r* | -  -  - | -  -  - | -  -  - | -  -  - | 0.0929  2.20  0.74 | 0.0224_(s)_  -3.62  0.88 | -  -  - | -  -  - |
| *w_1_^13^* | *p*  *t*_(4)_  *r* | 0.0185  3.84  0.89 | 2.19×10^-4^_(s)_  -12.73  0.99 | -  -  - | -  -  - | -  -  - | -  -  - | -  -  - | -  -  - |
| *w_2_^13^* | *p*  *t*_(4)_  *r* | 0.172  1.66  0.64 | 0.0018_(s)_  -7.36  0.97 | -  -  - | -  -  - | -  -  - | -  -  - | -  -  - | -  -  - |
| *w_3_^13^* | *p*  *t*_(4)_  *r* | 0.0252  3.49  0.87 | 3.68×10^-4^_(s)_  -11.15  0.98 | -  -  - | -  -  - | -  -  - | -  -  - | -  -  - | -  -  - |
| *w_4_^13^* | *p*  *t*_(4)_  *r* | 0.286  -1.23  0.52 | 2.58×10^-4^_(s)_  -12.21  0.99 | -  -  - | -  -  - | -  -  - | -  -  - | -  -  - | -  -  - |
| *w_5_^13^* | *p*  *t*_(4)_  *r* | 0.176  1.64  0.63 | 0.0023_(s)_  -6.92  0.96 | -  -  - | -  -  - | **-**  **-**  **-** | -  -  - | -  -  - | -  -  - |
| *w_6_^13^* | *p*  *t*_(4)_  *r* | 0.631  0.52  0.25 | 2.14×10^-5^_(s)_  -22.95  1.00 | -  -  - | -  -  - | **-**  **-**  **-** | -  -  - | -  -  - | -  -  - |

| ID | | Sub. I | | Sub. J | |
| --- | --- | --- | --- | --- | --- |
| *MNIIA*_VAF_ | | 0.548 | | 0.679 | |
| Criterion | | (1) | (2) | (1) | (2) |
| *w_1_^7^* | *p*  *t*_(4)_  *r* | 0.0011  8.30  0.97 | 0.182  -1.61  0.63 | 0.0277  3.39  0.86 | 0.007_(s)_  -5.10  0.93 |
| *w_1_^8^* | *p*  *t*_(4)_  *r* | 0.191  1.57  0.62 | 0.0018_(s)_  -7.41  0.97 | 0.031  3.26  0.85 | 0.0085_(s)_  -4.82  0.92 |
| *w_2_^8^* | *p*  *t*_(4)_  *r* | 8.03×10^-4^_(s)_  -9.12  0.98 | 4.28×10^-5^_(s)_  -19.26  0.99 | -  -  - | -  -  - |
| *w_3_^8^* | *p*  *t*_(4)_  *r* | 7.43×10^-5^  16.76  0.99 | 0.0238  3.55  0.87 | -  -  - | -  -  - |
| *w_1_^9^* | *p*  *t*_(4)_  *r* | 0.126  1.93  0.69 | 2.31×10^-5^_(s)_  -22.49  1.00 | 0.344  -1.07  0.47 | 1.06×10^-4^_(s)_  -15.33  0.99 |
| *w_2_^9^* | *p*  *t*_(4)_  *r* | 0.0078_(s)_  -4.95  0.93 | 1.82×10^-5^_(s)_  -23.90  1.00 | 0.014  4.18  0.90 | 0.0697  -2.46  0.78 |
| *w_3_^9^* | *p*  *t*_(4)_  *r* | -  -  - | -  -  - | 0.0654  2.52  0.78 | 0.0083_(s)_  -4.86  0.92 |
| *w_1_^10^* | *p*  *t*_(4)_  *r* | 0.200  1.54  0.61 | 0.0073_(s)_  -5.05  0.93 | 0.381  0.98  0.44 | 9.81×10^-4^_(s)_  -8.65  0.97 |
| *w_2_^10^* | *p*  *t*_(4)_  *r* | 0.123  1.95  0.70 | 0.0085_(s)_  -4.82  0.92 | 0.0464  2.85  0.82 | 1.08×10^-4^_(s)_  -15.25  0.99 |
| *w_3_^10^* | *p*  *t*_(4)_  *r* | -  -  - | -  -  - | 0.0742  2.40  0.77 | 0.0968  -2.16  0.73 |
| *w_1_^11^* | *p*  *t*_(4)_  *r* | 0.841  0.21  0.11 | 0.0392_(s)_  -3.02  0.83 | 0.838  0.22  0.11 | 0.0188_(s)_  -3.82  0.89 |
| *w_2_^11^* | *p*  *t*_(4)_  *r* | 0.0356  -3.12  0.84 | 7.61×10^-6^_(s)_  -29.74  1.00 | 0.0737  -2.41  0.77 | 4.30×10^-5^_(s)_  -19.24  0.99 |
| *w_3_^11^* | *p*  *t*_(4)_  *r* | 0.002_(s)_  -7.14  0.96 | 1.05×10^-5^_(s)_  -27.40  1.00 | 0.056  2.67  0.80 | 0.0024_(s)_  -6.81  0.96 |
| *w_1_^12^* | *p*  *t*_(4)_  *r* | 0.205  1.51  0.60 | 1.03×10^-4^_(s)_  -15.44  0.99 | 0.0107  4.51  0.91 | 2.00×10^-5^_(s)_  -23.33  1.00 |
| *w_2_^12^* | *p*  *t*_(4)_  *r* | 0.367  1.02  0.45 | 2.08×10^-4^_(s)_  -12.91  0.99 | 0.308  1.17  0.50 | 0.0738  -2.41  0.77 |
| *w_3_^12^* | *p*  *t*_(4)_  *r* | 0.526  0.69  0.33 | 1.17×10^-4^_(s)_  -14.93  0.99 | 7.42×10^-4^  9.30  0.98 | 0.0054_(s)_  -5.49  0.94 |

(b)

| ID | | Sub. A | | Sub. B | | Sub. C | | Sub. D | |
| --- | --- | --- | --- | --- | --- | --- | --- | --- | --- |
| *MNIIA*_CD_ | | 0.568 | | 0.596 | | 0.476 | | 0.488 | |
| Criterion | | (1) | (2) | (1) | (2) | (1) | (2) | (1) | (2) |
| *w_1_^5^* | *p*  *t*_(4)_  *r* | -  -  - | -  -  - | -  -  - | -  -  - | 0.0072  5.05  0.93 | 0.411  -0.92  0.42 | 0.456  -0.83  0.38 | 0.0248_(s)_  -3.50  0.87 |
| *w_2_^5^* | *p*  *t*_(4)_  *r* | -  -  - | -  -  - | -  -  - | -  -  - | -  -  - | -  -  - | 0.982  0.024  0.012 | 0.0167_(s)_  -3.96  0.89 |
| *w_1_^6^* | *p*  *t*_(4)_  *r* | -  -  - | -  -  - | 0.233  1.40  0.57 | 0.0126_(s)_  -4.30  0.91 | 0.0046  5.74  0.94 | 0.0635  -2.55  0.79 | 4.48×10^-4^  10.60  0.98 | 0.0487  2.80  0.81 |
| *w_1_^6^* | *p*  *t*_(4)_  *r* | -  -  - | -  -  - | 0.0023  6.93  0.96 | 0.411  0.92  0.42 | 0.0053  5.51  0.94 | 0.006_(s)_  -5.32  0.94 | 0.0021  7.11  0.96 | 8.69×10^-5^_(s)_  -16.11  0.99 |
| *w_1_^7^* | *p*  *t*_(4)_  *r* | 0.0019  7.31  0.96 | 0.102  2.11  0.73 | 0.517  0.71  0.33 | 4.94×10^-5^_(s)_  -18.58  0.99 | 0.0805  2.33  0.76 | 0.0261  -3.45  0.87 | 0.0033  6.26  0.95 | 0.266  -1.29  0.54 |
| *w_2_^7^* | *p*  *t*_(4)_  *r* | 0.0279  3.38  0.86 | 0.473  -0.79  0.37 | 0.834  -0.22  0.11 | 4.81×10^-5^_(s)_  -18.70  0.99 | -  -  - | -  -  - | -  -  - | -  -  - |
| *w_1_^8^* | *p*  *t*_(4)_  *r* | 0.112  2.03  0.71 | 0.195  -1.55  0.61 | 0.0044  5.80  0.95 | 0.368  1.02  0.45 | 0.0107  4.51  0.91 | 1.53×10^-4^_(s)_  -13.95  0.99 | 0.633  0.52  0.25 | 0.0707  -2.45  0.77 |
| *w_2_^8^* | *p*  *t*_(4)_  *r* | -  -  - | -  -  - | 0.05  2.78  0.81 | 6.05×10^-4^_(s)_  -9.81  0.98 | -  -  - | -  -  - | 0.186  1.60  0.62 | 0.0432  -2.92  0.83 |
| *w_3_^8^* | *p*  *t*_(4)_  *r* | -  -  - | -  -  - | 0.0142  4.15  0.90 | 0.665  0.47  0.23 | -  -  - | -  -  - | -  -  - | -  -  - |
| *w_4_^8^* | *p*  *t*_(4)_  *r* | -  -  - | -  -  - | 0.0316  -3.24  0.85 | 1.76×10^-4^_(s)_  -13.46  0.99 | -  -  - | -  -  - | -  -  - | -  -  - |
| *w_1_^9^* | *p*  *t*_(4)_  *r* | 0.021  3.69  0.88 | 0.475  -0.79  0.37 | 0.293  1.21  0.52 | 0.0068_(s)_  -5.15  0.93 | 0.0132  4.25  0.90 | 0.0046_(s)_  -5.73  0.94 | 0.0091  4.73  0.92 | 0.432  0.87  0.40 |
| *w_2_^9^* | *p*  *t*_(4)_  *r* | 0.881  0.16  0.08 | 0.0061_(s)_  -5.29  0.94 | 0.0061  5.31  0.94 | 2.77×10^-4^_(s)_  -12.00  0.99 | -  -  - | -  -  - | 0.031  3.26  0.85 | 0.779  -0.30  0.15 |
| *w_3_^9^* | *p*  *t*_(4)_  *r* | -  -  - | -  -  - | -  -  - | -  -  - | -  -  - | -  -  - | 0.079  2.34  0.76 | 0.0594  -2.61  0.79 |
| *w_4_^9^* | *p*  *t*_(4)_  *r* | -  -  - | -  -  - | -  -  - | -  -  - | -  -  - | -  -  - | 0.871  0.17  0.086 | 0.006_(s)_  -5.33  0.94 |
| *w_1_^10^* | *p*  *t*_(4)_  *r* | 0.0064  5.22  0.93 | 0.464  0.81  0.37 | 0.0035  6.16  0.95 | 0.125  1.93  0.70 | 7.57×10^-4^  9.26  0.98 | 0.193  1.56  0.62 | 0.0027  6.64  0.96 | 0.865  0.18  0.091 |
| *w_2_^10^* | *p*  *t*_(4)_  *r* | 0.0028  6.55  0.96 | 0.509  0.72  0.34 | 0.553  0.65  0.31 | 0.0521  -2.74  0.81 | 0.826  0.23  0.12 | 0.0073_(s)_  -5.03  0.93 | 0.0051  5.58  0.94 | 0.0327  -3.21  0.85 |
| *w_3_^10^* | *p*  *t*_(4)_  *r* | 0.0022  7.02  0.96 | 0.535  0.68  0.32 | 0.0018  7.36  0.97 | 0.96  0.05  0.03 | 0.0031  6.40  0.95 | 0.0067_(s)_  -5.17  0.93 | 0.005  5.58  0.94 | 0.0256  -3.47  0.87 |
| *w_4_^10^* | *p*  *t*_(4)_  *r* | 0.0056  5.42  0.94 | 0.0437  -2.91  0.82 | -  -  - | -  -  - | -  -  - | -  -  - | 0.0639  -2.54  0.79 | 4.81×10^-5^_(s)_  -18.71  0.99 |
| *w_1_^11^* | *p*  *t*_(4)_  *r* | 0.0771  2.37  0.76 | 0.232  -1.41  0.58 | 0.0643  2.54  0.79 | 0.184  -1.61  0.63 | 1.83×10^-4^  13.32  0.99 | 0.0126_(s)_  -4.30  0.91 | 0.149  1.79  0.67 | 0.222  -1.44  0.59 |
| *w_2_^11^* | *p*  *t*_(4)_  *r* | -  -  - | -  -  - | 0.318  1.14  0.50 | 0.0026_(s)_  -6.68  0.96 | -  -  - | -  -  - | 0.0037  6.06  0.95 | 0.995  -0.0067  0.0034 |
| *w_3_^11^* | *p*  *t*_(4)_  *r* | -  -  - | -  -  - | 0.002  7.16  0.96 | 0.782  -0.30  0.15 | -  -  - | -  -  - | 0.0052  5.52  0.94 | 0.0017_(s)_  -7.43  0.97 |
| *w_4_^11^* | *p*  *t*_(4)_  *r* | -  -  - | -  -  - | -  -  - | -  -  - | -  -  - | -  -  - | 2.64×10^-4^  12.14  0.99 | 0.0324  3,22  0.85 |
| *w_1_^12^* | *p*  *t*_(4)_  *r* | 0.0060  5.32  0.94 | 0.0102_(s)_  -4.58  0.92 | 0.273  1.27  0.54 | 5.35×10^-4^_(s)_  -10.13  0.98 | 0.231  1.41  0.58 | 0.0843  -2.29  0.75 | 2.45×10^-5^  22.18  1.00 | 0.0059  5.35  0.94 |
| *w_2_^12^* | *p*  *t*_(4)_  *r* | 0.0186  3.83  0.89 | 0.3038  -1.18  0.51 | -  -  - | -  -  - | 0.026  3.45  0.87 | 0.0023_(s)_  -6.90  0.96 | -  -  - | -  -  - |
| *w_3_^12^* | *p*  *t*_(4)_  *r* | -  -  - | -  -  - | -  -  - | -  -  - | 0.0496  2.78  0.81 | 0.018_(s)_  -3.87  0.89 | -  -  - | -  -  - |
| *w_1_^13^* | *p*  *t*_(4)_  *r* | -  -  - | -  -  - | -  -  - | -  -  - | 0.0669  2.50  0.78 | 4.96×10^-4^_(s)_  -10.33  0.98 | -  -  - | -  -  - |
| *w_2_^13^* | *p*  *t*_(4)_  *r* | -  -  - | -  -  - | -  -  - | -  -  - | 0.513  -0.72  0.34 | 0.0065_(s)_  -5.20  0.93 | -  -  - | -  -  - |
| *w_3_^13^* | *p*  *t*_(4)_  *r* | -  -  - | -  -  - | -  -  - | -  -  - | 0.0025  6.73  0.96 | 0.0418  -2.95  0.83 | -  -  - | -  -  - |
| *w_4_^13^* | *p*  *t*_(4)_  *r* | -  -  - | -  -  - | -  -  - | -  -  - | 2.96×10^-5^  21.13  1.00 | 5.58×10^-5^_(s)_  -18.02  0.99 | -  -  - | -  -  - |
| *w_5_^13^* | *p*  *t*_(4)_  *r* | -  -  - | -  -  - | -  -  - | -  -  - | 4.36×10^-4^  10.67  0.98 | 0.0053_(s)_  -5.52  0.94 | -  -  - | -  -  - |

| ID | | Sub. E | | Sub. F | | Sub. G | | Sub. H | |
| --- | --- | --- | --- | --- | --- | --- | --- | --- | --- |
| *MNIIA*_CD_ | | 0.468 | | 0.644 | | 0.635 | | 0.670 | |
| Criterion | | (1) | (2) | (1) | (2) | (1) | (2) | (1) | (2) |
| *w_1_^7^* | *p*  *t*_(4)_  *r* | 0.562  0.63  0.30 | 2.72×10^-4^_(s)_  -12.05  0.99 | 0.673  -0.46  0.22 | 0.0015_(s)_  -7.68  0.97 | 0.0367  3.09  0.84 | 0.792  -0.28  0.14 | 0.0035  6.19  0.95 | 0.158  -1.73  0.65 |
| *w_2_^7^* | *p*  *t*_(4)_  *r* | -  -  - | -  -  - | -  -  - | -  -  - | 0.167  1.69  0.64 | 0.181  -1.62  0.63 | -  -  - | -  -  - |
| *w_1_^8^* | *p*  *t*_(4)_  *r* | 0.0031  6.39  0.95 | 0.145  1.81  0.67 | 0.441  -0.86  0.39 | 0.184  -1.61  0.63 | 0.397  -0.95  0.43 | 0.001_(s)_  -8.52  0.97 | 0.0017  7.46  0.97 | 0.242  1.37  0.57 |
| *w_2_^8^* | *p*  *t*_(4)_  *r* | -  -  - | -  -  - | 0.966  -0.045  0.023 | 0.0063_(s)_  -5.25  0.93 | 0.0224  3.62  0.88 | 0.0499  -2.78  0.81 | 0.115  2.01  0.71 | 3.00×10^-6^_(s)_  -37.57  1.00 |
| *w_3_^8^* | *p*  *t*_(4)_  *r* | -  -  - | -  -  - | 0.747  0.35  0.17 | 0.0305  -3.28  0.85 | 0.0041  5.91  0.95 | 0.941  0.079  0.039 | -  -  - | -  -  - |
| *w_1_^9^* | *p*  *t*_(4)_  *r* | 0.974  -0.035  0.017 | 0.0048_(s)_  -5.67  0.94 | 0.858  0.19  0.095 | 0.0684  -2.48  0.78 | 0.408  0.92  0.42 | 0.0484  -2.81  0.81 | 0.0937  2.19  0.74 | 0.0229_(s)_  -3.59  0.87 |
| *w_2_^9^* | *p*  *t*_(4)_  *r* | -  -  - | -  -  - | 0.312  1.16  0.50 | 0.0165_(s)_  -3.97  0.89 | 0.174  1.65  0.64 | 0.0104_(s)_  -4.55  0.92 | 0.0028  6.56  0.96 | 0.0064_(s)_  -5.24  0.93 |
| *w_3_^9^* | *p*  *t*_(4)_  *r* | -  -  - | -  -  - | 0.959  0.055  0.028 | 0.0134_(s)_  -4.23  0.90 | -  -  - | -  -  - | -  -  - | -  -  - |
| *w_4_^9^* | *p*  *t*_(4)_  *r* | -  -  - | -  -  - | 0.304  1.18  0.51 | 0.0023_(s)_  -6.92  0.96 | -  -  - | -  -  - | -  -  - | -  -  - |
| *w_1_^10^* | *p*  *t*_(4)_  *r* | 0.760  -0.33  0.16 | 0.0404  -2.99  0.83 | 0.503  0.74  0.35 | 0.04  -3.00  0.83 | 0.0617  -2.57  0.79 | 0.002_(s)_  -7.21  0.96 | 0.150  1.78  0.66 | 7.79×10^-5^_(s)_  -16.56  0.99 |
| *w_2_^10^* | *p*  *t*_(4)_  *r* | 0.187  1.59  0.62 | 0.943  0.077  0.038 | 0.162  1.71  0.65 | 0.0044_(s)_  -5.79  0.95 | 0.111  2.04  0.71 | 0.0569  -2.65  0.80 | 0.0073  5.03  0.93 | 0.981  -0.026  0.013 |
| *w_3_^10^* | *p*  *t*_(4)_  *r* | 0.136  -1.86  0.68 | 0.0024_(s)_  -6.80  0.96 | -  -  - | -  -  - | -  -  - | -  -  - | -  -  - | -  -  - |
| *w_1_^11^* | *p*  *t*_(4)_  *r* | 0.0755  -2.39  0.77 | 0.0036_(s)_  -6.12  0.95 | 0.0087  4.79  0.92 | 0.862  -0.19  0.093 | 0.868  -0.18  0.088 | 0.0278  -3.38  0.86 | 0.0296  3.31  0.86 | 0.644  -0.50  0.24 |
| *w_2_^11^* | *p*  *t*_(4)_  *r* | 3.76×10^-5^  19.90  1.00 | 3.80×10^-5^_(s)_  -19.85  1.00 | 0.747  0.35  0.17 | 0.0448  -2.88  0.82 | 0.007  5.10  0.93 | 0.153  -1.76  0.66 | 0.226  1.43  0.58 | 0.007  -5.09  0.93 |
| *w_3_^11^* | *p*  *t*_(4)_  *r* | 0.0032  6.32  0.95 | 0.165  1.70  0.65 | -  -  - | -  -  - | -  -  - | -  -  - | 0.536  0.68  0.32 | 0.0018  -7.35  0.96 |
| *w_1_^12^* | *p*  *t*_(4)_  *r* | -  -  - | -  -  - | -  -  - | -  -  - | 0.0037  6.09  0.95 | 0.443  -0.85  0.39 | -  -  - | -  -  - |
| *w_2_^12^* | *p*  *t*_(4)_  *r* | -  -  - | -  -  - | -  -  - | -  -  - | 0.225  1.43  0.58 | 3.76×10^-6^_(s)_  -35.50  1.00 | -  -  - | -  -  - |
| *w_3_^12^* | *p*  *t*_(4)_  *r* | -  -  - | -  -  - | -  -  - | -  -  - | 0.128  1.92  0.69 | 0.043  -2.93  0.83 | -  -  - | -  -  - |
| *w_1_^13^* | *p*  *t*_(4)_  *r* | 0.0418  2.95  0.83 | 5.89×10^-4^_(s)_  -9.88  0.98 | -  -  - | -  -  - | -  -  - | -  -  - | -  -  - | -  -  - |
| *w_2_^13^* | *p*  *t*_(4)_  *r* | 0.220  1.45  0.59 | 0.0053_(s)_  -5.51  0.94 | -  -  - | -  -  - | -  -  - | -  -  - | -  -  - | -  -  - |
| *w_3_^13^* | *p*  *t*_(4)_  *r* | 0.051  2.76  0.81 | 9.50×10^-5^_(s)_  -8.73  0.97 | -  -  - | -  -  - | -  -  - | -  -  - | -  -  - | -  -  - |
| *w_4_^13^* | *p*  *t*_(4)_  *r* | 0.0048_(s)_  -5.65  0.94 | 4.40×10^-4^_(s)_  -10.65  0.98 | -  -  - | -  -  - | -  -  - | -  -  - | -  -  - | -  -  - |
| *w_5_^13^* | *p*  *t*_(4)_  *r* | 0.273  -1.27  0.54 | 0.0041_(s)_  -5.93  0.95 | -  -  - | -  -  - | **-**  **-**  **-** | -  -  - | -  -  - | -  -  - |
| *w_6_^13^* | *p*  *t*_(4)_  *r* | 0.0932  -2.19  0.74 | 3.50×10^-5^_(s)_  -20.26  1.00 | -  -  - | -  -  - | **-**  **-**  **-** | -  -  - | -  -  - | -  -  - |

| ID | | Sub. I | | Sub. J | |
| --- | --- | --- | --- | --- | --- |
| *MNIIA*_CD_ | | 0.476 | | 0.502 | |
| Criterion | | (1) | (2) | (1) | (2) |
| *w_1_^7^* | *p*  *t*_(4)_  *r* | 0.0016  7.67  0.97 | 0.571  -0.62  0.29 | 0.0414  2.96  0.83 | 0.0714  -2.44  0.77 |
| *w_1_^8^* | *p*  *t*_(4)_  *r* | 0.433  0.87  0.40 | 0.0053_(s)_  -5.51  0.94 | 0.0323  3.22  0.85 | 0.0268  -3.42  0.86 |
| *w_2_^8^* | *p*  *t*_(4)_  *r* | 4.11×10^-4^_(s)_  -10.84  0.98 | 7.16×10^-5^_(s)_  -16.91  0.99 | -  -  - | -  -  - |
| *w_3_^8^* | *p*  *t*_(4)_  *r* | 8.18×10^-5^  16.36  0.99 | 0.0069  5.11  0.93 | -  -  - | -  -  - |
| *w_1_^9^* | *p*  *t*_(4)_  *r* | 0.0043_(s)_  -5.82  0.95 | 4.18×10^-5^_(s)_  -19.38  0.99 | 0.200  -1.54  0.61 | 2.54×10^-4^_(s)_  -12.26  0.99 |
| *w_2_^9^* | *p*  *t*_(4)_  *r* | 0.0022_(s)_  -7.01  0.96 | 2.64×10^-5^_(s)_  -21.76  1.00 | 0.110  2.05  0.72 | 0.467  -0.80  0.37 |
| *w_3_^9^* | *p*  *t*_(4)_  *r* | -  -  - | -  -  - | 0.0682  2.48  0.78 | 0.0302  -3.29  0.85 |
| *w_1_^10^* | *p*  *t*_(4)_  *r* | 0.444  0.85  0.39 | 0.011_(s)_  -4.48  0.91 | 0.0493  -2.79  0.81 | 0.0029_(s)_  -6.47  0.96 |
| *w_2_^10^* | *p*  *t*_(4)_  *r* | 0.665  0.47  0.23 | 0.0138_(s)_  -4.19  0.90 | 0.0275  3.39  0.86 | 7.90×10^-4^_(s)_  -9.16  0.98 |
| *w_3_^10^* | *p*  *t*_(4)_  *r* | -  -  - | -  -  - | 0.224  1.44  0.58 | 0.230  -1.42  0.58 |
| *w_1_^11^* | *p*  *t*_(4)_  *r* | 0.516  -0.71  0.33 | 0.0614  -2.58  0.79 | 0.863  0.18  0.092 | 0.0720  -2.43  0.77 |
| *w_2_^11^* | *p*  *t*_(4)_  *r* | 0.0103_(s)_  -4.56  0.92 | 1.04×10^-5^_(s)_  -27.48  1.00 | 0.0463  -2.85  0.82 | 1.21×10^-4^_(s)_  -14.83  0.99 |
| *w_3_^11^* | *p*  *t*_(4)_  *r* | 3.44×10^-4^_(s)_  -11.34  0.98 | 1.33×10^-5^_(s)_  -25.83  1.00 | 0.0751  2.39  0.77 | 0.0079_(s)_  -4.92  0.93 |
| *w_1_^12^* | *p*  *t*_(4)_  *r* | 0.123  -1.95  0.70 | 1.70×10^-4^_(s)_  -13.58  0.99 | 0.0054  5.47  0.94 | 1.12×10^-4^_(s)_  -15.11  0.99 |
| *w_2_^12^* | *p*  *t*_(4)_  *r* | 0.599  -0.57  0.27 | 3.06×10^-4^_(s)_  -11.70  0.99 | 0.299  1.19  0.51 | 0.278  -1.25  0.53 |
| *w_3_^12^* | *p*  *t*_(4)_  *r* | 0.659  -0.48  0.23 | 1.74×10^-4^_(s)_  -13.51  0.99 | 9.59×10^-4^  8.70  0.97 | 0.0767  -2.37  0.76 |

(c)

| ID | | Sub. A | | Sub. B | | Sub. C | | Sub. D | |
| --- | --- | --- | --- | --- | --- | --- | --- | --- | --- |
| *MNIIA*_AD_ | | 0.608 | | 0.451 | | 0.588 | | 0.497 | |
| Criterion | | (1) | (2) | (1) | (2) | (1) | (2) | (1) | (2) |
| *w_1_^5^* | *p*  *t*_(4)_  *r* | -  -  - | -  -  - | -  -  - | -  -  - | 0.0086  4.81  0.92 | 0.179  -1.63  0.63 | 0.149  -1.79  0.67 | 0.0228_(s)_  -3.60  0.87 |
| *w_2_^5^* | *p*  *t*_(4)_  *r* | -  -  - | -  -  - | -  -  - | -  -  - | -  -  - | -  -  - | 0.86  0.19  0.094 | 0.0159_(s)_  -4.02  0.90 |
| *w_1_^6^* | *p*  *t*_(4)_  *r* | -  -  - | -  -  - | 0.339  1.09  0.48 | 0.309  -1.16  0.50 | 0.0062  5.26  0.93 | 0.0234_(s)_  -3.57  0.87 | 0.0026  6.66  0.96 | 0.0578  2.64  0.80 |
| *w_1_^6^* | *p*  *t*_(4)_  *r* | -  -  - | -  -  - | 0.0027  6.62  0.96 | 0.0707  2.45  0.77 | 0.0053  5.49  0.94 | 0.0026_(s)_  -6.66  0.96 | 0.152  1.77  0.66 | 7.68×10^-5^_(s)_  -16.62  0.99 |
| *w_1_^7^* | *p*  *t*_(4)_  *r* | 0.0020  7.17  0.96 | 0.238  1.39  0.57 | 0.344  -1.07  0.47 | 2.44×10^-4^_(s)_  -12.39  0.99 | 0.099  2.14  0.73 | 0.0082_(s)_  -4.87  0.92 | 0.0078  4.95  0.93 | 0.237  -1.39  0.57 |
| *w_2_^7^* | *p*  *t*_(4)_  *r* | 0.0326  3.21  0.85 | 0.327  -1.12  0.49 | 0.0042_(s)_  -5.89  0.95 | 1.45×10^-4^_(s)_  -14.13  0.99 | -  -  - | -  -  - | -  -  - | -  -  - |
| *w_1_^8^* | *p*  *t*_(4)_  *r* | 0.129  1.91  0.69 | 0.142  -1.82  0.67 | 0.0049  5.63  0.94 | 0.0429  2.93  0.83 | 0.0169  3.94  0.89 | 9.35×10^-5^_(s)_  -15.81  0.99 | 0.557  0.64  0.30 | 0.0679  -2.48  0.78 |
| *w_2_^8^* | *p*  *t*_(4)_  *r* | -  -  - | -  -  - | 0.337  -1.09  0.48 | 0.0037_(s)_  -6.06  0.95 | -  -  - | -  -  - | 0.232  1.41  0.58 | 0.409  -2.98  0.83 |
| *w_3_^8^* | *p*  *t*_(4)_  *r* | -  -  - | -  -  - | 0.0405  2.99  0.83 | 0.171  1.67  0.64 | -  -  - | -  -  - | -  -  - | -  -  - |
| *w_4_^8^* | *p*  *t*_(4)_  *r* | -  -  - | -  -  - | 8.60×10^-4^_(s)_  -8.96  0.98 | 3.33×10^-4^_(s)_  -11.44  0.99 | -  -  - | -  -  - | -  -  - | -  -  - |
| *w_1_^9^* | *p*  *t*_(4)_  *r* | 0.023  3.59  0.87 | 0.302  -1.19  0.51 | 0.358  1.04  0.46 | 0.0212_(s)_  -3.68  0.88 | 0.0250  3.49  0.87 | 0.0012_(s)_  -8.23  0.97 | 0.0124  4.33  0.91 | 0.509  0.72  0.34 |
| *w_2_^9^* | *p*  *t*_(4)_  *r* | 0.983  0.02  0.01 | 0.0046_(s)_  -5.73  0.94 | 0.0476  2.83  0.82 | 6.74×10^-4^_(s)_  -9.54  0.99 | -  -  - | -  -  - | 0.0296  3.31  0.86 | 0.739  -0.36  0.18 |
| *w_3_^9^* | *p*  *t*_(4)_  *r* | -  -  - | -  -  - | -  -  - | -  -  - | -  -  - | -  -  - | 0.145  1.81  0.67 | 0.0558  -2.67  0.80 |
| *w_4_^9^* | *p*  *t*_(4)_  *r* | -  -  - | -  -  - | -  -  - | -  -  - | -  -  - | -  -  - | 0.713  -0.40  0.19 | 0.0057_(s)_  -5.39  0.94 |
| *w_1_^10^* | *p*  *t*_(4)_  *r* | 0.0064  5.23  0.93 | 0.732  0.37  0.18 | 0.004  5.95  0.95 | 0.0153  4.06  0.90 | 0.037  3.08  0.84 | 0.962  -0.051  0.026 | 0.0026  6.68  0.96 | 0.974  0.034  0.017 |
| *w_2_^10^* | *p*  *t*_(4)_  *r* | 0.0028  6.53  0.96 | 0.955  0.06  0.03 | 0.611  0.55  0.27 | 0.104  -2.10  0.72 | 0.998  -0.0023  0.0012 | 0.0044_(s)_  -5.81  0.95 | 0.01  4.61  0.92 | 0.0292_(s)_  -3.33  0.86 |
| *w_3_^10^* | *p*  *t*_(4)_  *r* | 0.0021  7.11  0.96 | 0.792  0.28  0.14 | 0.0022  7.00  0.96 | 0.272  1.27  0.54 | 0.0036  6.14  0.95 | 0.0017_(s)_  -7.46  0.97 | 0.0095  4.68  0.92 | 0.0232_(s)_  -3.58  0.87 |
| *w_4_^10^* | *p*  *t*_(4)_  *r* | 0.0057  5.41  0.94 | 0.0269  -3.42  0.86 | -  -  - | -  -  - | -  -  - | -  -  - | 0.0213_(s)_  -3.68  0.88 | 4.67×10^-5^_(s)_  -18.84  0.99 |
| *w_1_^11^* | *p*  *t*_(4)_  *r* | 0.0755  2.39  0.77 | 0.147  -1.80  0.67 | 0.115  2.01  0.71 | 0.542  -0.67  0.32 | 5.12×10^-4^  10.24  0.98 | 0.0033_(s)_  -6.25  0.95 | 0.258  1.32  0.55 | 0.203  -1.52  0.61 |
| *w_2_^11^* | *p*  *t*_(4)_  *r* | -  -  - | -  -  - | 0.532  0.68  0.32 | 0.021_(s)_  -3.69  0.88 | -  -  - | -  -  - | 0.0201  3.74  0.88 | 0.938  -0.082  0.041 |
| *w_3_^11^* | *p*  *t*_(4)_  *r* | -  -  - | -  -  - | 0.0021  7.09  0.96 | 0.234  1.40  0.57 | -  -  - | -  -  - | 0.0041  5.89  0.95 | 0.0016_(s)_  -7.56  0.97 |
| *w_4_^11^* | *p*  *t*_(4)_  *r* | -  -  - | -  -  - | -  -  - | -  -  - | -  -  - | -  -  - | 0.0018  7.38  0.97 | 0.0351  3.13  0.84 |
| *w_1_^12^* | *p*  *t*_(4)_  *r* | 0.0060  5.32  0.94 | 0.0066_(s)_  -5.18  0.93 | 0.261  1.31  0.55 | 0.0019_(s)_  -7.23  0.96 | 0.248  1.35  0.56 | 0.0189_(s)_  -3.81  0.89 | 3.93×10^-5^  19.67  0.99 | 0.007  5.10  0.93 |
| *w_2_^12^* | *p*  *t*_(4)_  *r* | 0.0188  3.82  0.89 | 0.215  -1.47  0.59 | -  -  - | -  -  - | 0.877  -0.16  0.082 | 9.32×10^-4^_(s)_  -8.77  0.97 | -  -  - | -  -  - |
| *w_3_^12^* | *p*  *t*_(4)_  *r* | -  -  - | -  -  - | -  -  - | -  -  - | 0.761  0.33  0.16 | 0.0075_(s)_  -5.00  0.93 | -  -  - | -  -  - |
| *w_1_^13^* | *p*  *t*_(4)_  *r* | -  -  - | -  -  - | -  -  - | -  -  - | 0.387  0.97  0.44 | 2.58×10^-4^_(s)_  -12.21  0.99 | -  -  - | -  -  - |
| *w_2_^13^* | *p*  *t*_(4)_  *r* | -  -  - | -  -  - | -  -  - | -  -  - | 0.469  -0.80  0.36 | 0.0038_(s)_  -6.02  0.95 | -  -  - | -  -  - |
| *w_3_^13^* | *p*  *t*_(4)_  *r* | -  -  - | -  -  - | -  -  - | -  -  - | 0.0272  3.40  0.86 | 0.0126_(s)_  -4.30  0.91 | -  -  - | -  -  - |
| *w_4_^13^* | *p*  *t*_(4)_  *r* | -  -  - | -  -  - | -  -  - | -  -  - | 3.46×10^-4^  11.33  0.98 | 2.13×10^-4^_(s)_  -22.98  1.00 | -  -  - | -  -  - |
| *w_5_^13^* | *p*  *t*_(4)_  *r* | -  -  - | -  -  - | -  -  - | -  -  - | 4.32×10^-4^  10.70  0.98 | 0.0019_(s)_  -7.31  0.96 | -  -  - | -  -  - |

| ID | | Sub. E | | Sub. F | | Sub. G | | Sub. H | |
| --- | --- | --- | --- | --- | --- | --- | --- | --- | --- |
| *MNIIA*_AD_ | | 0.311 | | 0.619 | | 0.618 | | 0.628 | |
| Criterion | | (1) | (2) | (1) | (2) | (1) | (2) | (1) | (2) |
| *w_1_^7^* | *p*  *t*_(4)_  *r* | 0.896  0.14  0.069 | 0.0011_(s)_  -8.37  0.97 | 0.543  -0.66  0.32 | 0.0017_(s)_  -7.49  0.97 | 0.0547  2.69  0.80 | 0.886  -0.15  0.076 | 0.0038  6.05  0.95 | 0.259  -1.32  0.55 |
| *w_2_^7^* | *p*  *t*_(4)_  *r* | -  -  - | -  -  - | -  -  - | -  -  - | 0.198  1.54  0.61 | 0.205  -1.51  0.60 | -  -  - | -  -  - |
| *w_1_^8^* | *p*  *t*_(4)_  *r* | 0.0032  6.33  0.95 | 0.0337  3.17  0.85 | 0.387  -0.97  0.44 | 0.203  -1.52  0.61 | 0.356  -1.04  0.46 | 0.0012_(s)_  -8.15  0.97 | 0.0037  6.07  0.95 | 0.141  1.83  0.68 |
| *w_2_^8^* | *p*  *t*_(4)_  *r* | -  -  - | -  -  - | 0.914  -0.12  0.058 | 0.007_(s)_  -5.10  0.93 | 0.0791  2.34  0.76 | 0.0619  -2.57  0.79 | 0.141  1.83  0.67 | 3.64×10^-6^_(s)_  -35.77  1.00 |
| *w_3_^8^* | *p*  *t*_(4)_  *r* | -  -  - | -  -  - | 0.727  0.37  0.18 | 0.0336  -3.18  0.85 | 0.0177  3.89  0.89 | 0.797  0.28  0.14 | -  -  - | -  -  - |
| *w_1_^9^* | *p*  *t*_(4)_  *r* | 0.834  -0.22  0.11 | 0.0196_(s)_  -3.77  0.88 | 0.778  0.30  0.15 | 0.0832  -2.30  0.75 | 0.670  0.46  0.22 | 0.0559  -2.67  0.80 | 0.322  1.13  0.49 | 0.0407  -2.98  0.83 |
| *w_2_^9^* | *p*  *t*_(4)_  *r* | -  -  - | -  -  - | 0.989  0.015  0.0076 | 0.0193_(s)_  -3.79  0.88 | 0.324  1.12  0.49 | 0.0116_(s)_  -4.42  0.91 | 0.0061  5.30  0.94 | 0.0089_(s)_  -4.77  0.92 |
| *w_3_^9^* | *p*  *t*_(4)_  *r* | -  -  - | -  -  - | 0.634  -0.51  0.25 | 0.0155_(s)_  -4.05  0.90 | -  -  - | -  -  - | -  -  - | -  -  - |
| *w_4_^9^* | *p*  *t*_(4)_  *r* | -  -  - | -  -  - | 0.357  1.04  0.46 | 0.0026_(s)_  -6.72  0.96 | -  -  - | -  -  - | -  -  - | -  -  - |
| *w_1_^10^* | *p*  *t*_(4)_  *r* | 0.537  -0.67  0.32 | 0.527  0.69  0.33 | 0.802  0.27  0.13 | 0.0489  -2.80  0.81 | 0.0489  -2.80  0.81 | 0.0021_(s)_  -7.07  0.96 | 0.182  1.61  0.63 | 9.82×10^-5^_(s)_  -15.61  0.99 |
| *w_2_^10^* | *p*  *t*_(4)_  *r* | 0.166  1.69  0.65 | 0.272  1.27  0.54 | 0.217  1.46  0.59 | 0.0052_(s)_  -5.53  0.94 | 0.286  1.23  0.52 | 0.0637  -2.54  0.79 | 0.0136  4.21  0.90 | 0.746  0.35  0.17 |
| *w_3_^10^* | *p*  *t*_(4)_  *r* | 0.098  -2.15  0.73 | 0.0103_(s)_  -4.57  0.92 | -  -  - | -  -  - | -  -  - | -  -  - | -  -  - | -  -  - |
| *w_1_^11^* | *p*  *t*_(4)_  *r* | 0.0672  -2.49  0.78 | 0.0094_(s)_  -4.68  0.92 | 0.0091  4.74  0.92 | 0.951  0.065  0.033 | 0.925  -0.10  0.050 | 0.0299  -3.30  0.86 | 0.0284  3.36  0.86 | 0.956  -0.059  0.030 |
| *w_2_^11^* | *p*  *t*_(4)_  *r* | 4.09×10^-5^  19.48  0.99 | 4.14×10^-4^_(s)_  -10.82  0.98 | 0.701  0.41  0.20 | 0.0504  -2.77  0.81 | 0.0071  5.08  0.93 | 0.179  -1.63  0.63 | 0.200  1.53  0.61 | 0.0088_(s)_  -4.77  0.92 |
| *w_3_^11^* | *p*  *t*_(4)_  *r* | 0.0032  6.30  0.95 | 0.0292  3.33  0.86 | -  -  - | -  -  - | -  -  - | -  -  - | 0.645  0.50  0.24 | 0.0021_(s)_  -7.09  0.96 |
| *w_1_^12^* | *p*  *t*_(4)_  *r* | -  -  - | -  -  - | -  -  - | -  -  - | 0.0045  5.75  0.94 | 0.547  -0.66  0.31 | -  -  - | -  -  - |
| *w_2_^12^* | *p*  *t*_(4)_  *r* | -  -  - | -  -  - | -  -  - | -  -  - | 0.841  0.21  0.11 | 4.09×10^-6^  -34.75  1.00 | -  -  - | -  -  - |
| *w_3_^12^* | *p*  *t*_(4)_  *r* | -  -  - | -  -  - | -  -  - | -  -  - | 0.153  1.76  0.66 | 0.0471  -2.84  0.82 | -  -  - | -  -  - |
| *w_1_^13^* | *p*  *t*_(4)_  *r* | 0.0365  3.09  0.84 | 0.0026_(s)_  -6.70  0.96 | -  -  - | -  -  - | -  -  - | -  -  - | -  -  - | -  -  - |
| *w_2_^13^* | *p*  *t*_(4)_  *r* | 0.216  1.47  0.59 | 0.0263_(s)_  -3.44  0.86 | -  -  - | -  -  - | -  -  - | -  -  - | -  -  - | -  -  - |
| *w_3_^13^* | *p*  *t*_(4)_  *r* | 0.0584  2.63  0.80 | 0.0038_(s)_  -6.03  0.95 | -  -  - | -  -  - | -  -  - | -  -  - | -  -  - | -  -  - |
| *w_4_^13^* | *p*  *t*_(4)_  *r* | 0.001_(s)_  -8.60  0.97 | 8.76×10^-4^_(s)_  -8.91  0.98 | -  -  - | -  -  - | -  -  - | -  -  - | -  -  - | -  -  - |
| *w_5_^13^* | *p*  *t*_(4)_  *r* | 0.119  -1.98  0.70 | 0.0085_(s)_  -4.83  0.92 | -  -  - | -  -  - | **-**  **-**  **-** | -  -  - | -  -  - | -  -  - |
| *w_6_^13^* | *p*  *t*_(4)_  *r* | 1.97×10^-4^_(s)_  -13.09  0.99 | 6.60×10^-5^_(s)_  -17.27  0.99 | -  -  - | -  -  - | **-**  **-**  **-** | -  -  - | -  -  - | -  -  - |

| ID | | Sub. I | | | Sub. J | |
| --- | --- | --- | --- | --- | --- | --- |
| *MNIIA*_AD_ | | 0.442 | | | 0.419 | |
| Criterion | | (1) | | (2) | (1) | (2) |
| *w_1_^7^* | *p*  *t*_(4)_  *r* | 0.0019  7.27  0.96 | | 0.898  -0.14  0.068 | 0.0743  2.40  0.77 | 0.295  -1.20  0.52 |
| *w_1_^8^* | *p*  *t*_(4)_  *r* | 0.325  -1.12  0.49 | | 0.0101_(s)_  -4.60  0.92 | 0.0327  3.21  0.85 | 0.0504  -2.77  0.81 |
| *w_2_^8^* | *p*  *t*_(4)_  *r* | 3.92×10^-4^_(s)_  -10.97  0.98 | | 9.41×10^-5^_(s)_  -15.79  0.99 | -  -  - | -  -  - |
| *w_3_^8^* | *p*  *t*_(4)_  *r* | 8.11×10^-5^  16.39  0.99 | | 0.0042  5.86  0.95 | -  -  - | -  -  - |
| *w_1_^9^* | *p*  *t*_(4)_  *r* | 5.59×10^-4^_(s)_  -10.01  0.98 | | 5.74×10^-5^_(s)_  -17.89  0.99 | 7.76×10^-4^_(s)_  -9.20  0.98 | 4.11×10^-4^_(s)_  -10.84  0.98 |
| *w_2_^9^* | *p*  *t*_(4)_  *r* | 9.68×10^-4^_(s)_  -8.68  0.97 | | 3.20×10^-5^_(s)_  -20.74  1.00 | 0.104  2.10  0.72 | 0.972  -0.037  0.019 |
| *w_3_^9^* | *p*  *t*_(4)_  *r* | -  -  - | | -  -  - | 0.554  0.65  0.31 | 0.0623  -2.56  0.79 |
| *w_1_^10^* | *p*  *t*_(4)_  *r* | 0.034  -3.16  0.85 | | 0.0137_(s)_  -4.20  0.90 | 0.0325  -3.21  0.85 | 0.0055_(s)_  -5.46  0.94 |
| *w_2_^10^* | *p*  *t*_(4)_  *r* | 0.617  0.54  0.26 | | 0.0176_(s)_  -3.89  0.89 | 0.357  -1.04  0.46 | 0.0032_(s)_  -6.33  0.95 |
| *w_3_^10^* | *p*  *t*_(4)_  *r* | -  -  - | | -  -  - | 0.183  1.61  0.63 | 0.344  -1.07  0.47 |
| *w_1_^11^* | *p*  *t*_(4)_  *r* | 0.290  -1.22  0.52 | | 0.0771  -2.37  0.76 | 0.954  0.061  0.031 | 0.149  -1.79  0.67 |
| *w_2_^11^* | *p*  *t*_(4)_  *r* | 0.0066_(s)_  -5.17  0.93 | | 1.23×10^-5^_(s)_  -26.39  1.00 | 0.0550  -2.68  0.80 | 2.16×10^-4^_(s)_  -12.78  0.99 |
| *w_3_^11^* | *p*  *t*_(4)_  *r* | 3.11×10^-4^_(s)_  -11.65  0.99 | | 1.50×10^-5^_(s)_  -25.08  1.00 | 0.0557  2.67  0.80 | 0.0154_(s)_  -4.05  0.90 |
| *w_1_^12^* | *p*  *t*_(4)_  *r* | 0.0385  -3.04  0.84 | | 2.22×10^-4^_(s)_  -12.69  0.99 | 0.0678  2.49  0.78 | 3.49×10^-4^_(s)_  -11.30  0.98 |
| *w_2_^12^* | *p*  *t*_(4)_  *r* | 0.292  -1.21  0.52 | | 3.73×10^-4^_(s)_  -11.11  0.98 | 0.313  1.15  0.50 | 0.512  -0.72  0.34 |
| *w_3_^12^* | *p*  *t*_(4)_  *r* | 0.0013_(s)_  -8.06  0.97 | | 2.12×10^-4^_(s)_  -12.84  0.99 | 9.18×10^-4^  8.81  0.98 | 0.406  -0.93  0.42 |
|  | | |  | | | |
